# Chitin- and Keratin-Rich Soil Amendments Suppress Rhizoctonia solani Disease via Changes to the Soil Microbial Community

**DOI:** 10.1128/AEM.00318-21

**Published:** 2021-05-11

**Authors:** Beatriz Andreo-Jimenez, Mirjam T. Schilder, Els H. Nijhuis, Dennis E. te Beest, Jaap Bloem, Johnny H. M. Visser, Gera van Os, Karst Brolsma, Wietse de Boer, Joeke Postma

**Affiliations:** a Biointeractions & Plant Health, Wageningen University & Research, Wageningen, The Netherlands; b Biometris, Wageningen University & Research, Wageningen, The Netherlands; c Animal Ecology, Wageningen University & Research, Wageningen, The Netherlands; d Field Crops, Wageningen University & Research, Wageningen, The Netherlands; e Aeres University of Applied Sciences, Dronten, The Netherlands; f Eurofins Agro, Wageningen, The Netherlands; g Department of Microbial Ecology, Netherlands Institute of Ecology, NIOO-KNAW, Wageningen, The Netherlands; h Soil Biology Group, Wageningen University & Research, Wageningen, The Netherlands; University of Illinois at Chicago

**Keywords:** *Rhizoctonia solani*, soil suppressiveness, compost, keratin, chitin, microbiome

## Abstract

Enhancing soil suppressiveness against plant pathogens or pests is a promising alternative strategy to chemical pesticides. Organic amendments have been shown to reduce crop diseases and pests, with chitin products the most efficient against fungal pathogens. To study which characteristics of organic products are correlated with disease suppression, an experiment was designed in which 10 types of organic amendments with different physicochemical properties were tested against the soilborne pathogen Rhizoctonia solani in sugar beet seedlings. Organic amendments rich in keratin or chitin reduced Rhizoctonia solani disease symptoms in sugar beet plants. The bacterial and fungal microbial communities in amended soils were distinct from the microbial communities in nonamended soil, as well as those in soils that received other nonsuppressive treatments. The *Rhizoctonia*-suppressive amended soils were rich in saprophytic bacteria and fungi that are known for their keratinolytic and chitinolytic properties (i.e., *Oxalobacteraceae* and *Mortierellaceae*). The microbial community in keratin- and chitin-amended soils was associated with higher zinc, copper, and selenium, respectively.

**IMPORTANCE** Our results highlight the importance of soil microorganisms in plant disease suppression and the possibility to steer soil microbial community composition by applying organic amendments to the soil.

## Abstract (precis)

Our results highlight the importance of soil microorganisms in plant disease suppression and the possibility to steer soil microbial community composition by applying organic amendments to the soil.

## INTRODUCTION

Crop losses due to plant diseases and pests are a problem worldwide, with average yield losses ranging from 17 to 30% in the five major food crops (i.e., wheat, rice, maize, potato, and soybean) (1, 2). Preventative measures are the most effect way to protect a crop against soilborne pathogens. However, some of these measures (e.g., the use of chemical soil fumigants) are banned or restricted due to environmental hazards. Creating disease-suppressive soils can be an effective strategy against multiple diseases (3, 4), and the addition of organic materials to the soil is one of the methods found to induce such suppressiveness (5, 6). In addition, organic amendments provide essential nutritional elements and can improve other soil quality parameters. As a result of the European Waste Framework Directive (Council Directive 2008/98/EC), more organic-rich waste products are promoted for agricultural use, which helps contribute to a circular economy (7, 8). Nowadays, a wide range of organic amendments with the potential to support plant health and improve nutrient use efficiency can be applied in agriculture (9).

Compost is the most commonly used organic amendment used to create enhanced soil suppressiveness against soilborne diseases (10–12), but it yields variable results, and the effects are case specific (5, 6). Chitin, a by-product from the seafood industry (crab, lobster, and shrimp shells), has been used since the 1970s to reduce damage by plant-parasitic nematodes (13–15) and fungal pathogens (15–18). Other organic waste products have been applied to control plant parasitic nematodes, such as oil cakes derived from several plant species, cellulosic waste, sugarcane bagasse, bone and horn meal, sewage sludge, manure, and various crop residues (19). Similarly, several plant- and animal-derived materials have been tested to reduce the detrimental effect of soilborne plant diseases (12, 18, 20–22). Although disease reduction has been demonstrated with several types of organic waste products, the effects are often inconsistent, and the level of control depends on the product, type of soil, plant-pathogen combination, and cultivation system (5, 6). Moreover, disease suppression via organic amendments operates through various mechanisms (6, 20, 23). However, most studies to date have focused on the organic amendment suppression mechanisms related to chemical structure and decomposition rate, rather than on effects mediated through the soil microbial community.

To better predict the effect of organic amendments on soil suppressiveness, the physicochemical properties of the organic products, as well as their impact on soil properties such as their microbial community, are being studied. For example, organic products have been characterized in terms of their chemical composition, pH, C/N ratio, and decomposition rate (9, 12). Other studies have assessed chemical, physical, and/or biological soil properties after being amended with organic products to determine possible links to disease suppression (24, 25). The use of organic amendments can affect soil microbial communities (26–28), and several recent studies have demonstrated correlations between soil microbial community composition and soil suppressiveness against plant pathogens (18, 22, 29). Therefore, integration of organic amendments and beneficial soil microorganisms might be the key for controlling soilborne pathogens (6).

The development of a tailor-made pipeline to select the best organic amendment and/or microbial consortium treatments to increase suppressiveness against a specific pathogen is of great interest to farmers. To achieve this, further insight into the mechanisms behind pathogen suppression in soil is needed. In order to better understand the mechanisms behind the stimulation of soil suppressiveness via the addition of organic products and which specific factors can be traced as disease suppressiveness markers, the following questions must be answered. (i) Which types of organic soil amendments have the potential to stimulate disease suppressiveness? (ii) Can the stimulation of disease suppression by organic amendments be ascribed to enrichment of specific microbial groups? (iii) Does enhanced disease suppression correlate with biological and physicochemical soil properties?

To address these questions, 10 organic products with different characteristics (i.e., variation in C/N ratio and decomposition rate) were selected to assess their potential to enhance disease suppression in soil. After two different soils from arable fields were amended with the products, pot experiments were performed to assess soil suppressiveness against the fungal pathogen Rhizoctonia solani. In addition, several soil characteristics were analyzed, as well as bacterial and fungal community composition and how microorganisms interact within these communities, to better understand the effect of the organic amendments on creating disease-suppressive soils.

## RESULTS

### Chitin- and keratin-rich organic amendments increase disease suppression.

**FIG 1:**
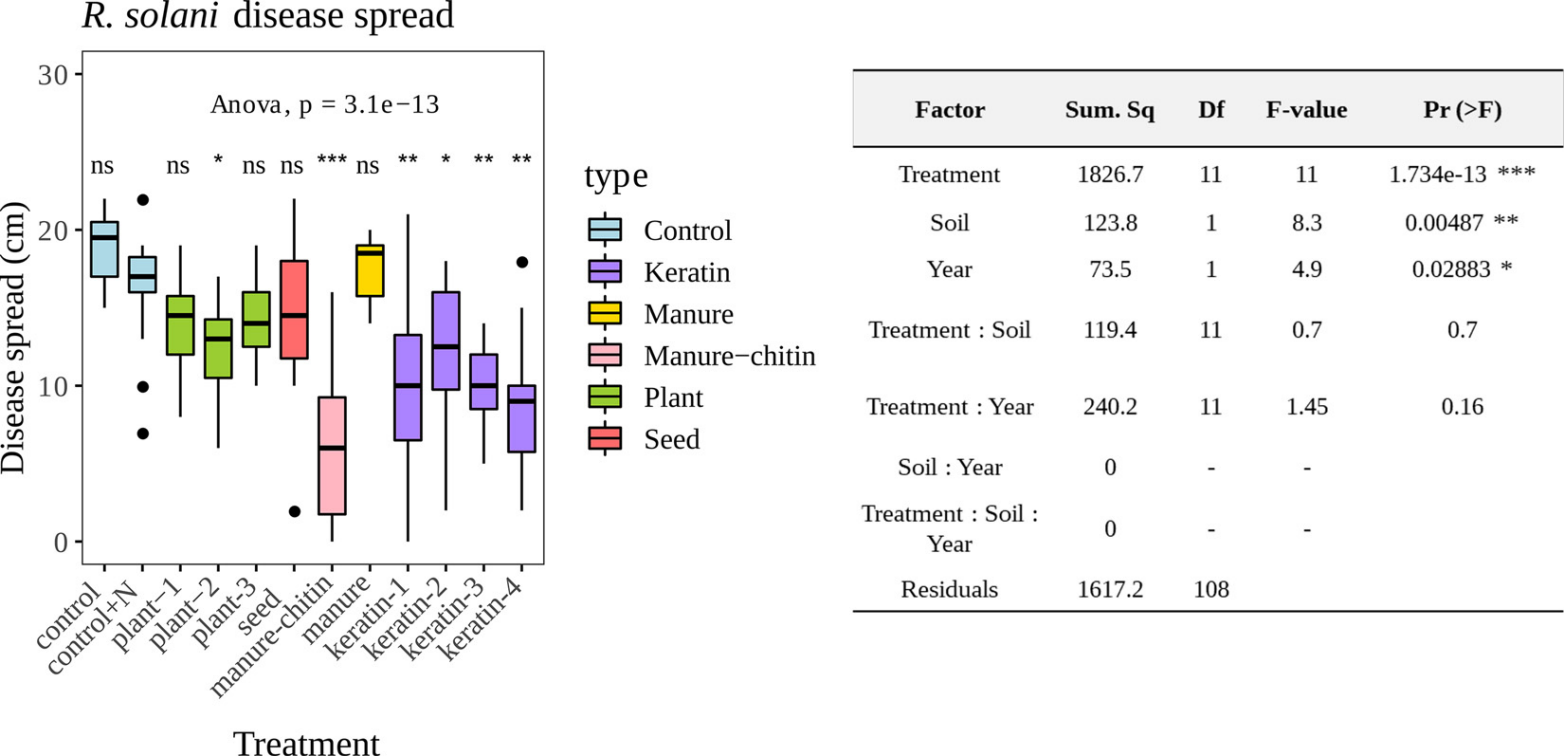
Disease spread from R. solani bioassays with sugar beet. Manure-chitin, plant-2, and all keratin-rich amendments reduced R. solani disease symptoms in plants. Disease scores are based on the maximal disease spread in centimeters from the inoculation point 21 days after pathogen inoculation. Colors are based on the type of amendment (Table 1). Asterisks represent the adjusted *P* values (*, *P* < 0.05; **, *P* < 0.01; ***, *P* < 0.001) from pairwise *t* test comparisons (Holm correction) using control+N as the reference. The table shows ANOVA results to test the effect of the different variables on disease suppression.

Bioassays with 10 different organic products (Table 1) added to two sandy soils were performed in 2016 and 2017 to assess the effect of these organic amendments on disease suppressiveness against R. solani in sugar beet seedlings. Two control treatments (i.e., soil with only nitrogen fertilizer [control+N] and nonamended soil [control]), were included. All data from both years and both soils were analyzed together to explore treatment effects on the target disease. Data from 2016 from Vredepeel soil were excluded from the data set as explained in the Materials and Methods. The amendments with plant-2, manure-chitin, and all four keratin products significantly reduced the disease spread compared to the control with an equal dosage of nitrogen (control+N) (Fig. 1). This demonstrates a disease-suppressive effect of these products independent of nitrogen concentration.

**TABLE 1:** Origins and applied dosages of organic amendment types

Treatment	Product description^*a*^	Amt of amended product (g/kg)^*b*^	Provider	Result for product^*c*^	
				C/N ratio	Respiration rate (nmol O_2_/kg OM/h)
Control	0
Control+N	Ca(NO_3_)_2_ fertilizer	1.2
Plant-1	Vegetable, fruit and garden waste composted	26.1	Vereniging Afvalbedrijven	11.9	5.4
Plant-2	Green waste composted	52.2	BVOR	16.3	6.0
Plant-3^*d*^	Ditch cuttings/ensiled grass	100.0/62.5	Waterschap Vallei/Veluwe	11.1/25.0	38.7/9.8
Seed	Defatted seed meal of *Brassica juncea* (Terrafit-Biofum)	3.8	P. H. Peterson Saatsucht Lundsgaard	8.8	7.3
Manure-chitin	Composted horse manure, chicken manure, gypsum, and water colonized with *Agaricus bisporus*	23.2	CNC	14.5	30.7
Manure	Granulated pig manure	9.3	Darling Ingredients	15.7	11.8
Keratin-1	Chicken feathers (Kerapro Son)	1.5	Darling Ingredients	3.4	26.4
Keratin-2	Chicken feathers (Kerapro Slow Release)	1.5	Darling Ingredients	3.6	21.2
Keratin-3	Pig hair	1.4	Darling Ingredients	3.3	24.2
Keratin-4	Chicken feathers and pig hair with bacterial biostimulant	1.5	Ecostyle BV	3.3	27.1

a Based on the main component.

b Amended product as g (fresh weight) per kg of soil. The final concentration was 0.2 g N/kg soil. The mean from both years is shown.

c Only for products. Shown are average values from 2016 and 2017.

d Product origins were different between the years 2016 and 2017.

### Biological and physicochemical soil properties differ among organic amendment treatments.

Organic amendments had different effects on the measured biological soil properties. Potentially mineralizable nitrogen (PMN), an indicator for labile nitrogen potentially available for plants, showed the most distinct changes across different organic amendments. PMN was increased 2 to 7 times by all organic amendments, except for plant-2, whereas the highest increase occurred with the seed treatment, a product made from defatted brassica seeds (see Fig. S1 in the supplemental material). On the other hand, hot water extractable carbon (HWC), an indicator of labile carbon, did not differ among most treatments: only in the case of manure-chitin (Fig. S1). Soil bacterial biomass was higher in all organic amendment treatments, with the exception of plant-1 and plant-2. Fungal biomass and ergosterol were highest in soil with seed and manure-chitin amendments (Fig. S1). Ergosterol is used as an indicator to quantify living fungal biomass because this lipid is almost exclusively found in fungal cell membranes. However, despite being related parameters, ergosterol and fungal biomass (based on hyphal length) presented different responses to some of the other amendments. For example, plant-3 and manure amendments presented higher ergosterol content, whereas the fungal biomass was not different from that of the control.

Many other physicochemical characteristics were quantified in the amended soils (see Fig. S2 and Table S1 in the supplemental material). All treatments showed a higher availability of copper (Cu.a) and total manganese (Mn.t) content compared with the control. Remarkably, potassium concentration (K.a and K.t) was higher in all amended soils, except in the case of keratin-rich amended soils, whose concentrations were the same as the control. The manure-chitin treatment had very high levels of available calcium and selenium (Ca.a and Se.a), along with higher molybdenum (Mo.a), which was also high in plant-based amendments (Fig. S2). Phosphate concentrations (P.a and P.t) were higher in manure treatments. The rest of the soil variables measured were not different among the treatments.

**FIG 2:**
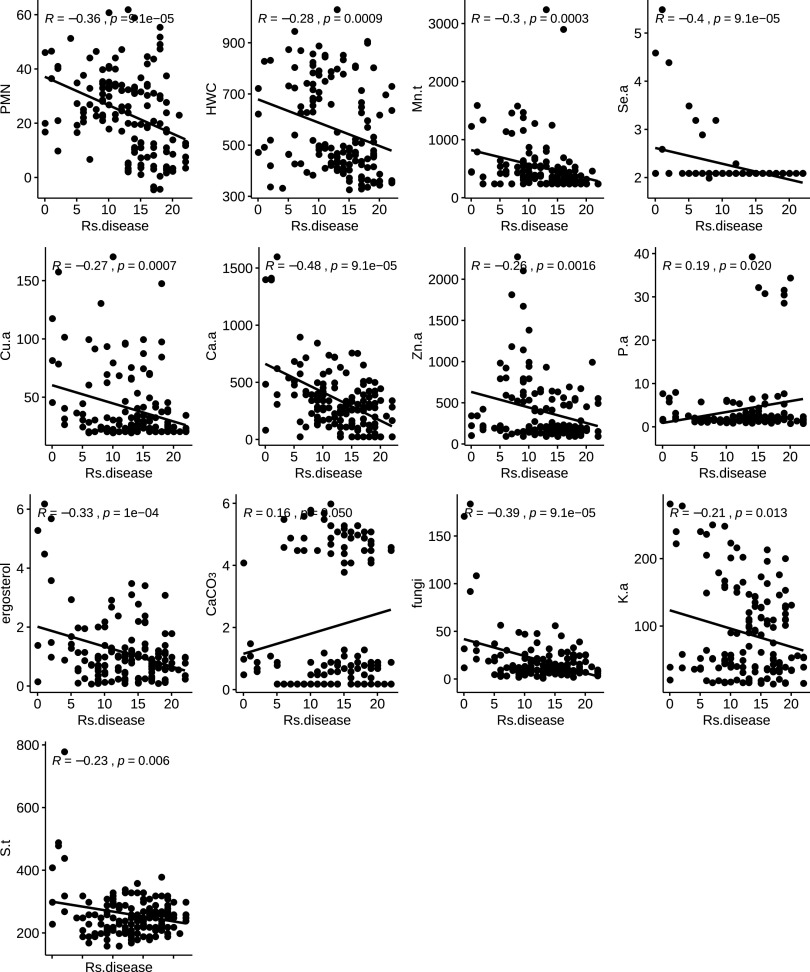
Correlation plots between soil parameters and R. solani disease spread. Only parameters that have a significant correlation with disease suppression are shown. PMN, potentially mineralizable nitrogen; HWC, hot water extractable carbon; Mn.t, total manganese; Se.a, available selenium; Cu.a, available copper; Ca.a, available calcium; Zn.a, available zinc; P.a, available phosphate; K.a, available potassium; S.t: total sulfur. *R* and *P* indicate Pearson correlation values and the permutation-based *P* values, respectively.

One of the objectives of this study was to find soil properties that correlated with disease suppression. To achieve this, we performed correlation analyses between all soil properties, concentrations, and R. solani disease spread (number of infected plants). The disease spread had a significant (*P* < 0.001) negative correlation with the biological soil properties PMN (Pearson, *R* = −0.36) and HWC (Pearson, *R* = −0.28), demonstrating that amended soil was more suppressive of R. solani at higher PMN and HWC values (Fig. 2). Other extremely negative correlations (*P* < 0.001) were between suppression of R. solani and chemical products such as total manganese (Mn.t) and selenium (Se.a) and available calcium (Ca.a), zinc (Zn.a), and copper (Cu.a). Furthermore, available potassium (K.a) and sulfur (S.a) (*P* < 0.01) were also negatively correlated with disease levels, but to a lesser degree. On the other hand, available phosphate (P.a) and calcium carbonate (CaCO_3_) showed a slight positive correlation with disease levels (*P* < 0.05) (Fig. 2).

Since the organic products had been selected for their variation in C/N ratios and respiration rates (Table 1; see supporting information at https://doi.org/10.4121/12971528), it was relevant to test if these properties can predict disease suppression after the soil was amended with these products. Interestingly, R. solani disease levels in amended soils correlated negatively with the respiration rate of the organic products (*R* = −0.8, *P* = 0.0045), whereas the C/N ratio of products showed no significant correlation with the disease (*R *= 0.5, *P* = 0.14).

### Bacterial and fungal microbial soil communities are different due to the organic amendments.

**FIG 3:**
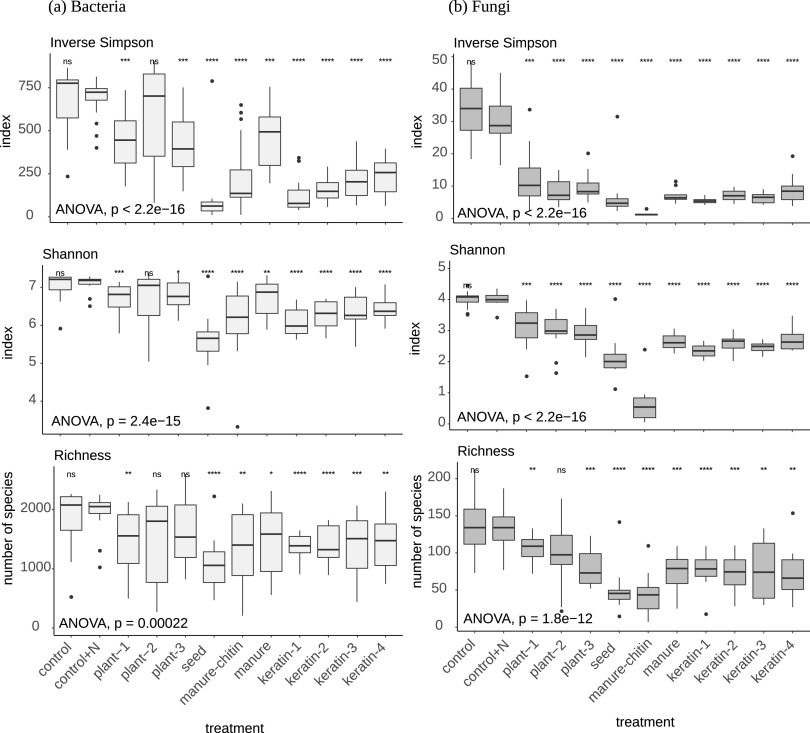
Microbial α diversity parameters in the study. Richness and inverse Simpson and Shannon diversity indices are shown for bacterial (a) and fungal (b) communities in the amended soils 3 weeks after mixing the organic products with the soil. The indices were produced using the rarefied abundance data. Asterisks represent the adjusted *P* values from pairwise *t* test comparisons (Holm correction) for all independent treatments using control+N as a reference. *, *P* < 0.05; **, *P* < 0.01; ***, *P* < 0.001; ns, not significant.

A total of 10,577,545 internal transcribed spacer (ITS) sequences (fungi) and 23,776,295 16S sequences (bacteria) were analyzed. These sequences were assigned to 1,383 and 15,750 fungal and bacterial amplicon sequence variants (ASVs), respectively (see supporting information at https://doi.org/10.4121/12971528). Three weeks after mixing organic products with the soil, microbial community structure was altered and community diversity was diminished (Shannon and inverse Simpson indices) compared to the nonamended and nitrogen fertilizer-amended controls (control and control+N) (Fig. 3). This was due to a lower number of species (richness) (Fig. 3). Bacterial diversity (Shannon diversity index [*H*′]) values ranged from around 7.5 in the controls, to 6.5 to 6.8 in keratin-amended soil, to 5.5 to 6.0 in seed-amended soil. Fungal diversity (Shannon diversity index [*H*′]) values were 4.0 in the controls, 2.5 to 3.0 in keratin-amended soil, and less than 0.5 in manure-chitin-amended soil. This extreme low fungal diversity in soil with manure-chitin is due to dominance of the inoculated Agaricus bisporus, which was added as active ingredient in the product amendments with plant-1, plant-2, and plant-3, and manure product had the least effect on bacterial diversity.

**FIG 4:**
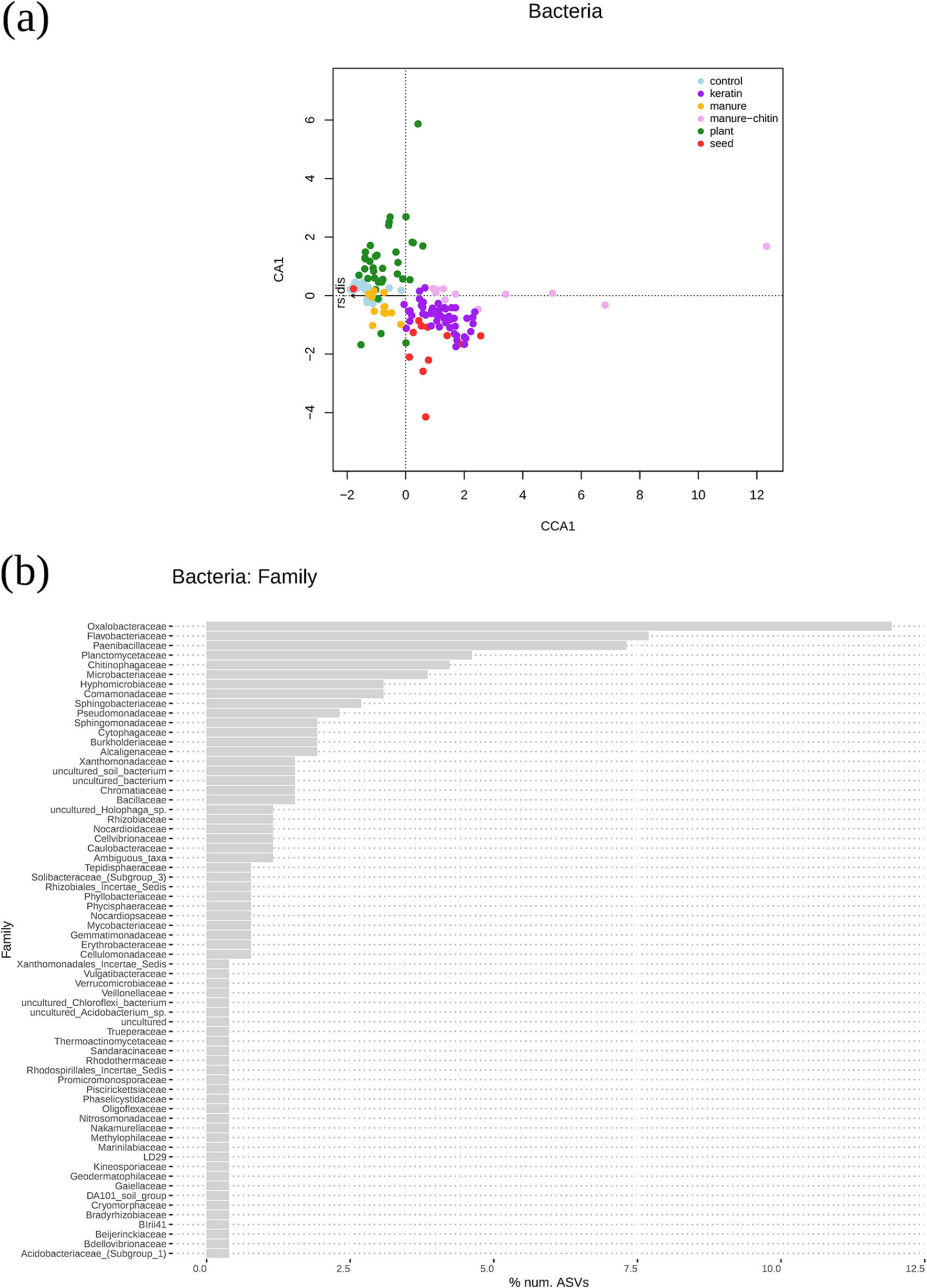

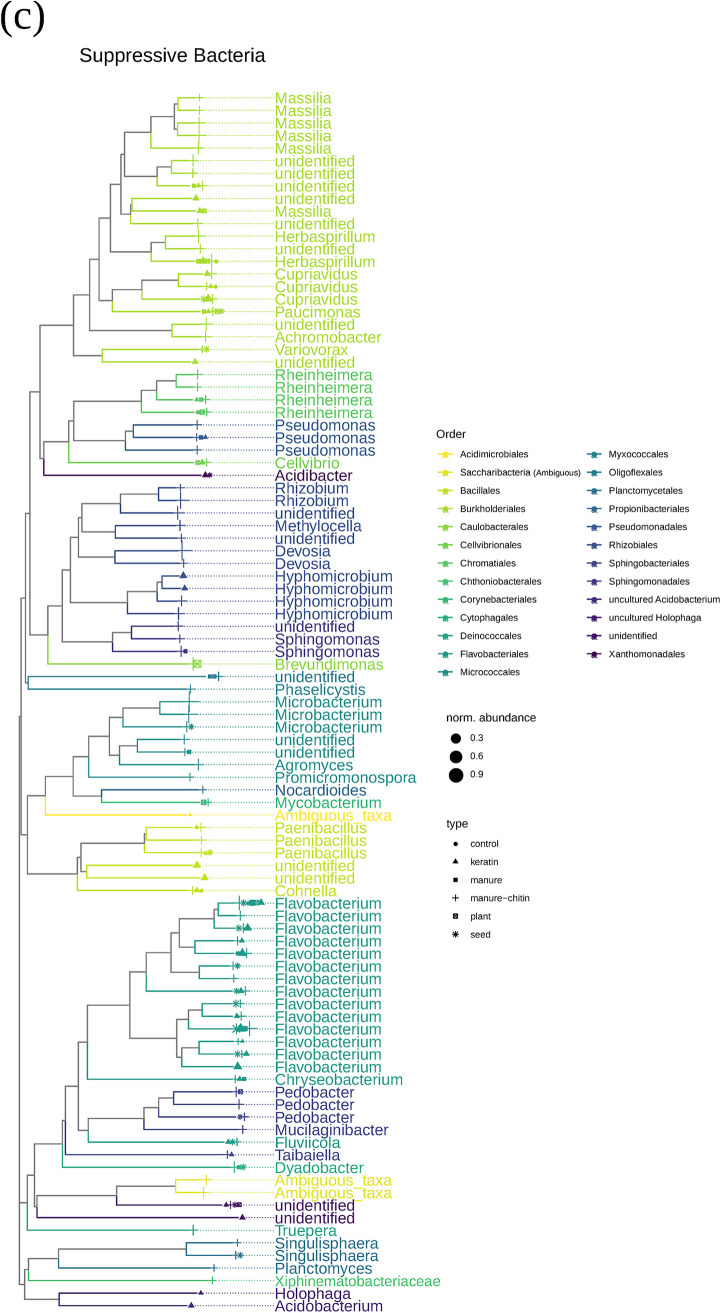
Bacterial community compositions in the different treatments. Potential disease-suppressive ASVs were selected based on the CCA using those more represented in manure-chitin and keratin treatments. (a) Plot representing CCA showing the microbiome differences between treatments and their relationship with R. solani disease (rs.dis). (b) Bar plot representing the percentage of the top 260 ASVs from the different family levels, which were closely related to manure-chitin and keratin treatments. (c) Phylogenetic tree that includes the top 100 ASVs from the most common groups in the chitin-manure and keratin treatments. Tip labels are genus names. The size of the symbol indicates the sum of normalized abundances for each ASV in the different treatments.

**FIG 5:**
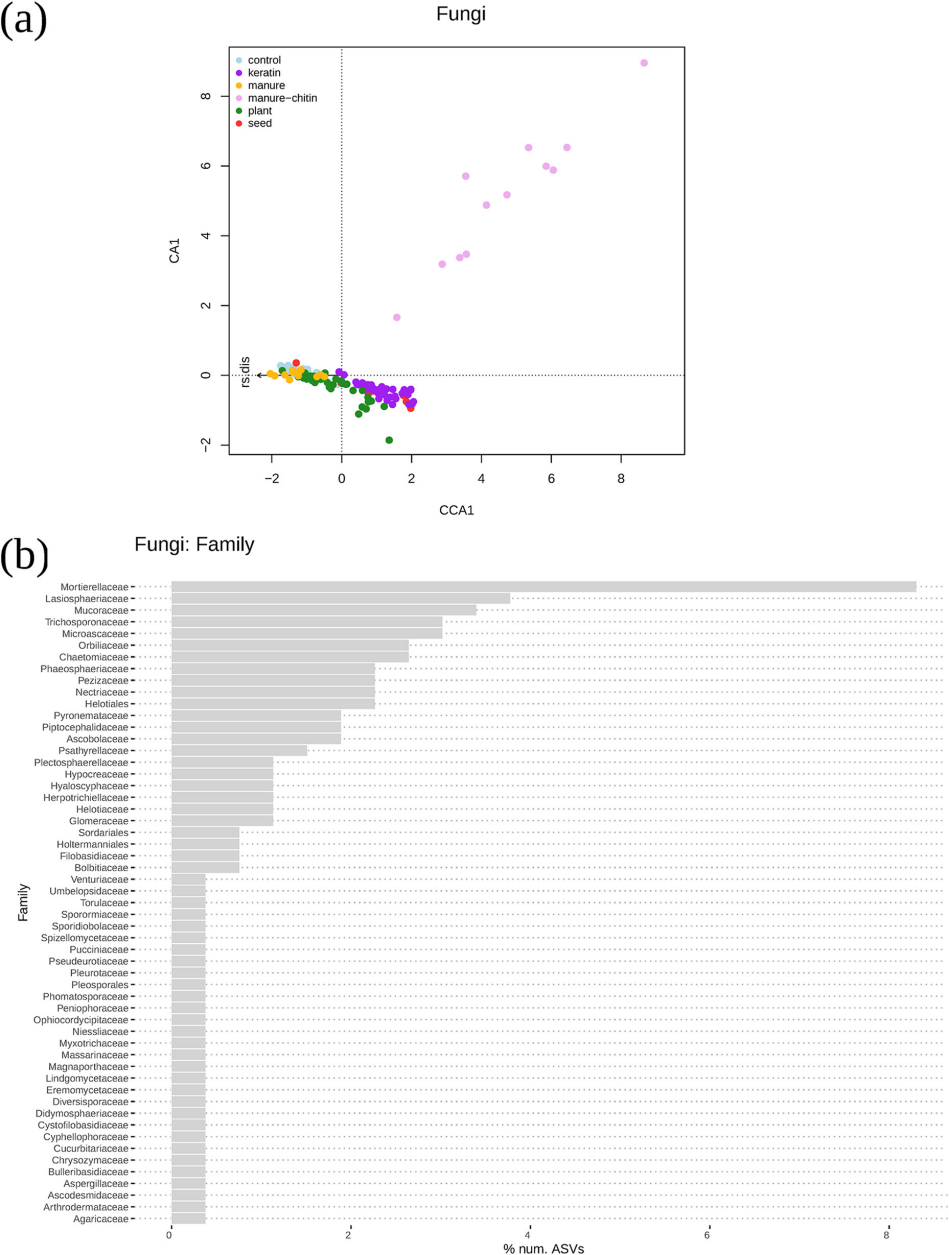

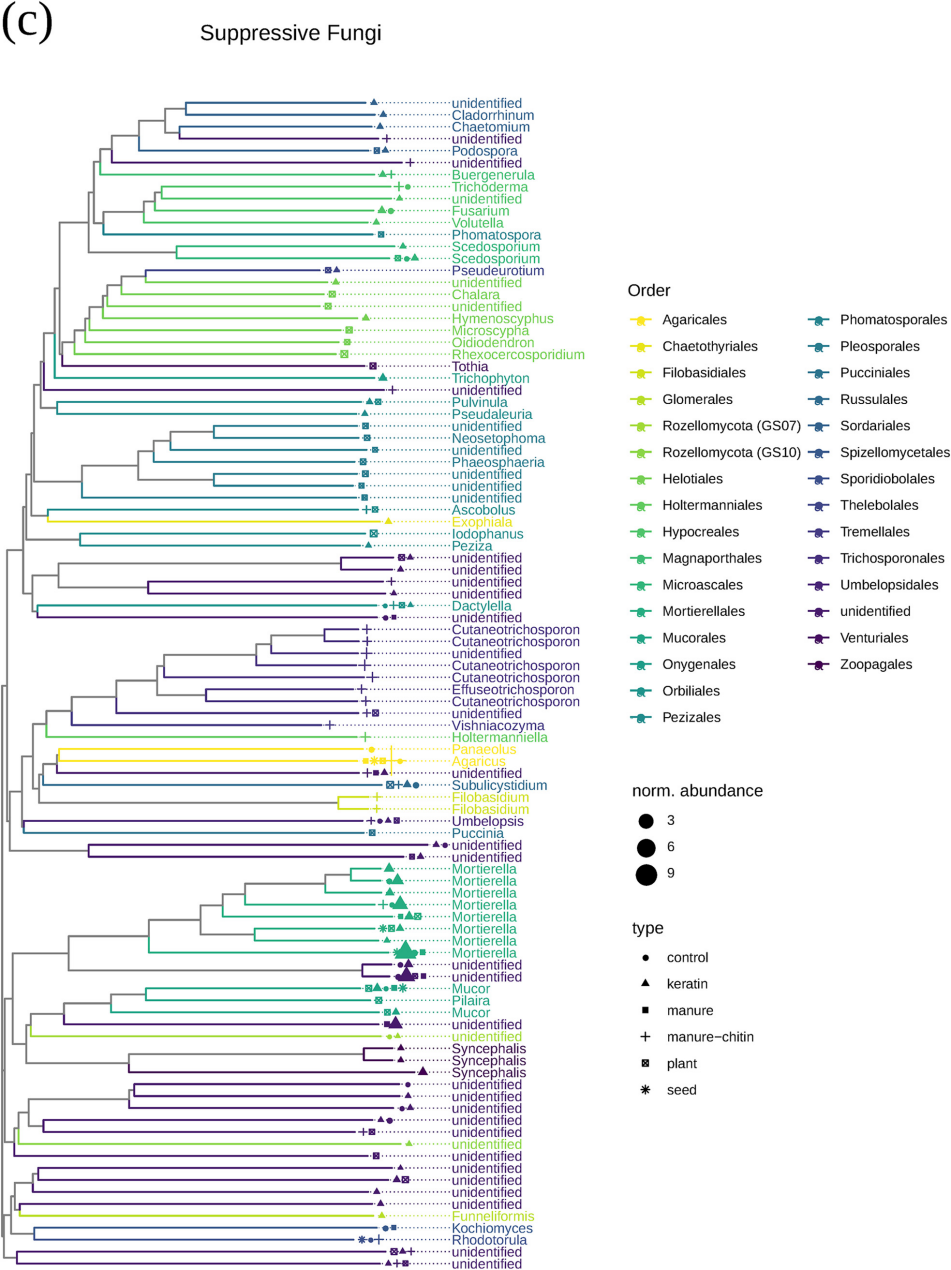
Fungal community compositions in the different treatments. Potential disease-suppressive ASVs were selected based on the CCA using those most represented in manure-chitin and keratin treatments. (a) Plot representing CCA showing the microbiome differences between treatments and their relationship with R. solani disease (rs.dis). (b) Bar plot representing the percentage of the top 260 ASVs number from the different family levels, which were closely related to manure-chitin and keratin treatments. (c) Phylogenetic tree that includes the top 100 ASVs from the most common groups in the chitin-manure and keratin treatments. Tip labels are genus names. The size of the symbols indicate the sum of normalized abundances for each ASV in the different treatments.

In order to study how the entire microbial community composition varied between soil amendments and years, a multivariate analysis (nonmetric multidimensional scaling [NMDS]) was performed. The soil microbial communities differed among types of soil and experimental years (see Fig. S4 in the supplemental material). Microbial community compositions were different between Lisse and Vredepeel soils for fungal (*adonis R*^2^ = 0.16, *P* < 0.001) and bacterial (*adonis R*^2^ = 0.26, *P* < 0.001) communities (see Fig. S3 in the supplemental material). Both microbial communities varied between the experiments in 2016 and 2017 as well. The type of soil had stronger effects than the treatments, and the year slightly affected microbial communities as well. However, it was possible to focus solely on the effects of the treatment on the microbial community composition and its correlation with disease suppression in a canonical correspondence analysis (CCA). Both fungal and bacterial microbial community compositions in the soils with control, plant, and manure treatments clustered together (Fig. 4 and 5). Furthermore, the four keratin treatments clustered together and partially overlapped with the soil microbial communities from seed-amended soil. The manure-chitin-amended samples presented the most distinct microbial community composition. Both the bacterial (*adonis R*^2^ = 0.46, *P* < 0.001) and fungal (*adonis R*^2^ = 0.51, *P* < 0.001) community compositions in the four keratin treatments and the manure-chitin treatment all together positively correlated with higher R. solani suppression (Fig. 4 and 5).

### *Oxalobacteraceae* and *Mortierellaceae* are more represented in suppressive treatments.

From the CCA, it was possible to select the fungal and bacterial taxa (ASVs) that were associated together with the most suppressive treatments (i.e., keratin and manure-chitin amendments) (see supporting information at https://doi.org/10.4121/12971528). The bacterial families *Oxalobacteraceae* (*Massilia* spp. and *Cupriavidus* spp.), *Flavobacteraceae* (*Flavobacterium* spp.), and *Paenibacillaceae* are the most represented taxa among the ASVs correlating strongest with R. solani disease suppression in comparison to the whole microbial community, and all three families were most abundant in chitin- and keratin-rich treatments (Fig. 4; Fig. S4). The manure-chitin amendment, which is composed of very different components compared to the other organic amendments, showed a unique bacterial community composition. Many of the ASVs that strongly associated with disease suppression (see the rank list of species in Data File 12 in the supporting information at https://doi.org/10.4121/12971528) were the most represented in manure-chitin-treated soil samples (i.e., *Massilia* [*Oxalobacteraceae*], *Microbacterium* [*Microbacteriaceae*], *Rhizobium* and *Devosia* [*Rhizobiaceae*]) (Fig. 4). On the other hand, *Flavobacterium* spp. (*Flavobacteraceae*) were more abundant or uniquely represented in keratin-amended soil samples (Fig. 4).

Among all selected fungal ASVs that correlated with R. solani disease suppression, *Mortierellaceae* was by far the most represented group, and it occurred almost exclusively in keratin-amended soils (Fig. 5; Fig. S4). A phylogenetic cluster of *Cutaneotrichosporon* spp. (Trichosporonaceae) and *Filobasidium* spp. (Filobasidiaceae) were only detected in the manure-chitin samples (Fig. 5). Furthermore, one ASV belonging to *Agaricus* spp. (Agaricaceae) was more abundant in the manure-chitin treatment (Fig. 5c), which is due to the inoculation of the product containing *Agaricus bisporus*.

To study what types of relationships were established between fungal and bacterial populations in R. solani disease-suppressive treatments, a network analysis was performed, where only the most abundant ASVs were included. Results showed three main fungal network hubs (i.e., *Panaeolus* spp., *Mortierella* spp., and an undescribed fungus from the order Chaetothyriales), which are connected to several bacterial ASVs belonging to different taxa (i.e., *Variovorax* [*Comamonadaceae*] and *Oxalobacteraceae*) (see Fig. S5 in the supplemental material). It is remarkable that the relationships are mainly negative, meaning that when the relative abundances of the fungi were reduced, the bacteria were relatively more abundant or vice versa.

### Specific soil chemical properties are associated with the microbial community in R. solani*-*suppressive treatments.

**FIG 6:**
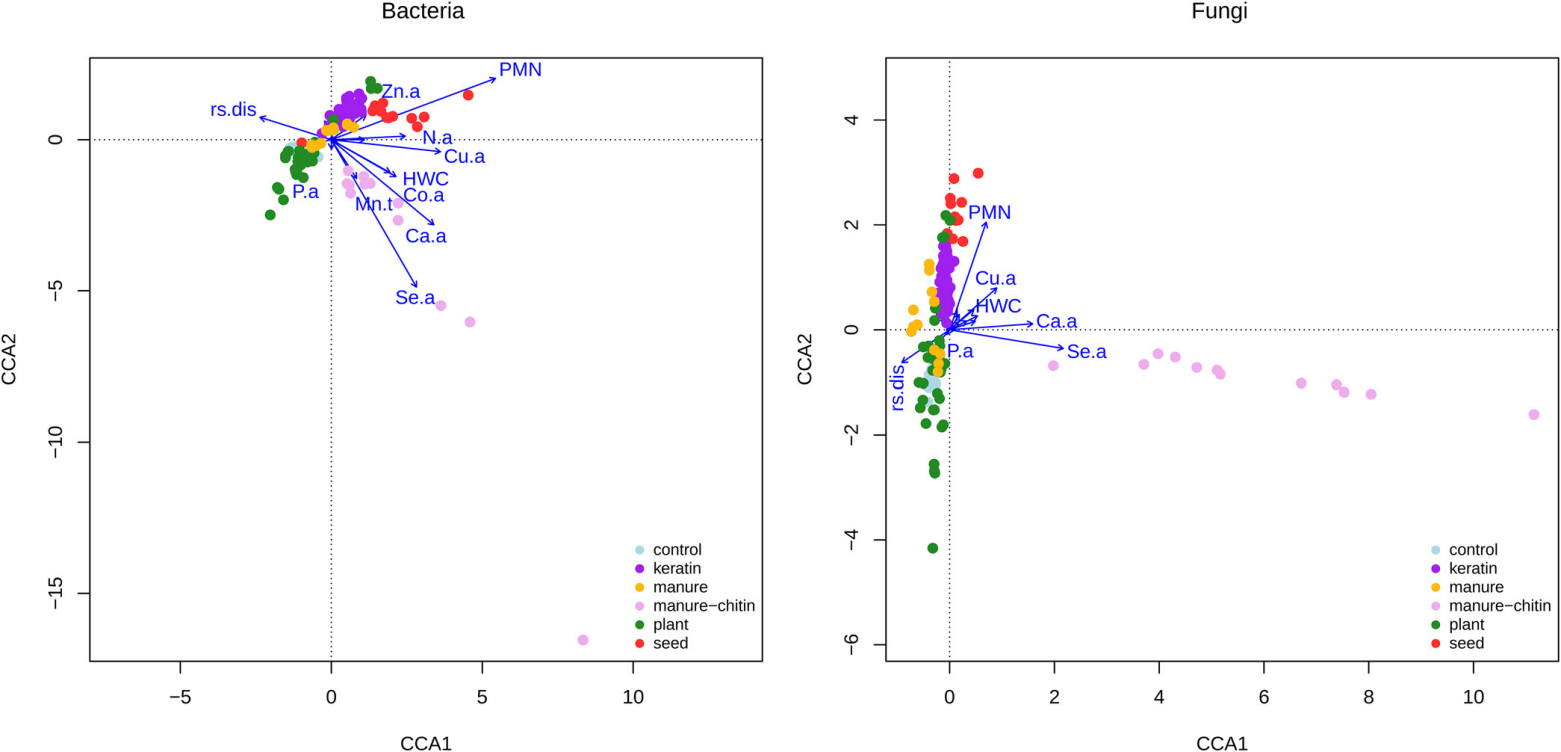
Associations between amended soil microbial communities and soil parameters. A CCA between microbial communities and soil parameters was performed. Copper (Cu.a), selenium (Se.a), and zinc (Zn.a) are positively associated with manure-chitin-rich and keratin-rich samples, respectively, in the bacterial (a) microbiome, while mainly copper (Cu.a) is associated with the fungal (b) microbial community for keratin-rich samples (based on ANOVA with manure-chitin-rich and keratin-rich treatments only [see Table S2 in the supplemental material]). All data except data presented in the Vredepeel 2016 data set are included. Soil parameters were selected based on their significant effect on disease suppression (from correlation analyses). Only the labels of parameters with the most significant effects on communities are shown. rs.dis, R. solani disease spread; HWC, hot water extractable carbon; PMN, potential mineralizable nitrogen; Zn.a, available zinc; N.a, available nitrogen; Cu.a, available copper; P.a, available phosphorous; Mn.t, total manganese; Co.a, available cobalt; Ca.a: available calcium; Se.a: available selenium.

Associations between the response of the soil microbial community and specific soil chemical properties to the organic treatments were detected. In manure-chitin samples, the bacterial community was associated with higher levels of copper (Cu.a) and selenium (Se.a) and marginally with available cobalt (Co.a) (Fig. 6; Table S1). On the other hand, in the keratin-rich-amended soils, fungal and bacterial microbial communities were linked to available zinc (Zn.a) and copper (Cu.a). Furthermore, for both treatments, microbial communities were associated with lower phosphate content (P.a) (Fig. 6; Table S1). Moreover, the microbial communities in treatments that did not reduce R. solani disease, such as manure and plant-3 amendments, were associated with higher carbon-to-nitrogen (C/N) ratios and available phosphorus (P.a).

## DISCUSSION

We found that chitin- and keratin-rich soil amendments play an important role in R. solani disease suppression. Furthermore, we showed that in soil treated with chitin- and keratin-rich amendments, the microbial community composition was very different from those of the nonsuppressive soils. Specifically, chitin- and keratin-rich amended soils were enriched in *Oxalobacteraceae* and *Mortierelleceae* species abundance. In those treatments, soil microbial communities related to disease suppression were positively correlated with zinc and copper, although the concentrations of these elements did not vary significantly across treatments. On the other hand, calcium and selenium concentrations were higher in the suppressive treatments. Based on these results, it might be possible to use specific microbial taxonomic groups and/or available calcium and selenium compounds as indicators for R. solani suppression in soils or as soil supplements to boost R. solani disease suppression.

We aimed to find explanatory factors for soil suppressiveness by using organic products with a wide range of properties and effects on disease suppression. A positive effect of keratin- and chitin-rich soil amendments on R. solani disease suppression was demonstrated in the current experiments, as well as in previous research (21). Suppressiveness was not induced by the nitrogen in these products, since the addition of mineral nitrogen [Ca(NO_3_)_2_] alone did not increase soil disease suppressiveness. Furthermore, the possibility that nitrogen in the organic products may be converted to other forms (e.g., NH_3_) that are toxic to some microbial species (30) was discarded.

We found that the microbial communities in all amended soils were different, with these differences becoming more apparent when treatments were grouped by amendment type (i.e., plant, manure, keratin, manure-chitin, seed). However, in some instances these differences in soil microbial community composition correlated with disease suppressiveness, and at other times they did not. It has been shown that chitin-enriched compost improves soil suppressive capacity against R. solani (31), where chitin- and keratin-degrading fungi (i.e., *Trichoderma*) are more abundant and act as active antagonists of R. solani. Furthermore, keratin-based products have shown plant growth promotion via increased activity of keratinolytic bacteria, which can act as antagonists to plant pathogens (32, 33). The keratin- and chitin-rich amendments in this study were enriched in chitinolytic/keratinolytic fungi such as *Cutaneotrichosporum* (Trichosporonales), *Trichoderma* (Hypocreales), *Trichophyton* (Onygenales), and *Mucor* (Mucorales). Although chitin and keratin are very different molecules (a glucosamine polymer and a protein, respectively), their degradation can be performed by the same microorganisms, as keratinolytic fungi are able to produce other hydrolytic enzymes such as chitinases (34). It can be hypothesized that the presence of high levels of keratin (as well as chitin) promotes the presence of both keratinolytic and chitinolytic fungi. The most abundant group found in the keratin-amended soils is Mortierellales. It has been reported that *Mortierella*, *Aspergillus*, and *Mucor* species have a chitinolytic activity (35). Moreover, *Mortierella* spp. have been reported for their capacity to degrade other toxic and recalcitrant compounds, to accumulate heavy metals such as zinc, and to induce the production of plant hormones such as indole-3-acetic acid (IAA) and abscisic acid (ABA), as well as antibiotics (36–38).

Some soil bacteria are able to degrade both chitin and keratin (35, 39), and some of them were more abundant in keratin- and chitin-rich amended soils in the present study. Bacteria such as *Flavobacterium*, *Pseudomonas*, *Microbacterium*, *Devosia* spp. (*Hyphomicrobiaceae*), and *Rhizobium* spp. (*Rhizobiaceae*), which were abundant in chitin-rich samples, have been shown to have pathogen (e.g., R. solani) suppression effects (40, 41). *Massilia* spp. (*Oxalobacteraceae*) were the dominant species in chitin-rich amended soils, and they were less abundant in soils treated with plant-derived amendments, which has also been observed in other studies (42). These species are an important component of the rhizosphere microbiota and they can thrive on the seed coat, radicle, and roots of plants, as well as on the hyphae of the pathogen *Pythium*, hence reducing its pathogenicity. Furthermore, *Massilia* is a well-known chitinolytic bacterium that is able to degrade keratin (43, 44). Because of its keratin degradation property, it is likely that it can thrive in keratin-rich environments and is able to reduce the growth of fungal pathogens as well.

In the present work, fungal and bacterial communities showed strong correlations. In R. solani-suppressive amended soils (keratin- and chitin-rich), there were clear interactions between fungal and bacterial soil communities. The abundance of *Mortierella* spp. negatively correlated with several bacterial taxa, among them an uncultured *Alcaligenaceae* taxon. Some bacteria belonging to this taxon (e.g., *Alcaligenes* spp.) are keratinase-producing bacteria (45).

One remarkable observation is that microbial diversity (inverse Simpson and Shannon indices) was lower in all amended soils and that the disease-suppressive treatments were among the soils with the lowest diversity indices, together with the seed-based amendment treatment (Terrafit-Biofum). Other microbial ecology studies support the importance of having a highly diverse microbial community to help create a better ecosystem and resilience (46). In the present study, we measured the short-term response (after 3 weeks) of the physicochemical soil properties and found that they were drastically changed by the amended products. This presumably allowed more opportunistic, fast-growing microbes to proliferate, as they are more adapted to carbon-enriched environments. It has been shown that disturbance reduces the microbial Shannon diversity index, as well as microbial community functions in agricultural environments (47). A highly diverse environment (in this case, the soil before treatment) is essential to provide the soil system with the plasticity to better respond against pathogens and/or other inputs. Future research is needed to focus on how long these changes in the microbial communities will persist.

Keratin- and chitin-rich amended soils had very distinct bacterial and fungal microbial communities compared to the other treatments. Furthermore, these microbial communities were linked to specific soil chemical properties (zinc and copper). Another factor, potential mineralizable nitrogen (PMN), which usually positively correlates with total microbial biomass (48), positively correlated with R. solani suppression. However, PMN was not associated with the microbial community composition in amended, suppressive soils. On the other hand, total fungal biomass was higher only in the chitin-rich amended soils that were colonized by *A. bisporus*. Selenium compounds are accumulated in *A. bisporus* hyphae and fruiting bodies (49, 50), which explains why selenium concentrations were highly positively correlated with the microbial communities in the chitin-rich treatment. Although zinc levels were not significantly different between treatments, zinc was positively correlated with microbial community structure in keratin-rich treatments. Therefore, it is suspected that positive correlations between zinc and disease suppression are the result of the ability of zinc to enhance specific microbial groups that reduce disease. This supports findings that zinc-enriched products have been shown to have a fungicidal effect on several soilborne pathogens (51, 52). Microbial keratin decomposition can be compared with that of other recalcitrant polymers such as cellulose and chitin. A specialized family of metalloenzymes that cleave cellulose and chitin, lytic polysaccharide monooxygenases (LPMOs), are produced by keratin-degrading fungi as well (e.g., *Trichoderma* and *Trichophyton* [39, 53]) and contain a metal binding site that can oxidize cellulose, hemicellulose, and chitin (54). We hypothesize that the observed higher zinc levels in keratin treatments might be due to higher production of specialized LPMOs containing a zinc binding site by keratinolytic microorganisms.

Bacterial taxa that have been correlated with R. solani disease suppression in former studies (29), such as *Massilia* spp., *Cupriavidus* spp., *Flavobacterium* spp., *Mortierella* spp., and *Cutaneotrichosporon* spp., could be used together with zinc and copper as markers for disease suppression. In addition, chitin-rich amendments have been more thoroughly studied, and their effects on soil quality and abiotic/biotic plant stress tolerance observed here are supported by past research (35, 55). Keratin-based amendments can likewise boost the plant-growth-promoting effect of symbiotic microorganisms such as Bacillus subtilis (32).

Despite the advances in knowledge about using soil amendments to enhance disease suppression presented in this study, still there are open questions. For example, is disease suppression due to enhancement of specific microbial functions? How long do changes to the soil microbial community composition and soil chemical properties last? Metatranscriptomic analyses could help to gain insight into the molecular functions behind the effects of amendments on R. solani disease spread by, for example, looking into the production and function of specialized enzymes that degrade chitin and keratin (e.g., LPMOs). Finally, the data presented here were collected at one time point. However, to better understand the duration of the effects of treatments on disease suppression, a time line study is needed, in which microbial populations and functions are tracked together with levels of disease suppression across multiple time points.

### Conclusions.

We showed that soil amendments rich in keratin and chitin are able to reduce R. solani disease in sugar beet. Furthermore, the microbial community is distinct in soils treated with different types of amendments (plant, seed, manure, keratin, and manure-chitin) and nonamended soils. Available forms of zinc and copper were related to soil microbial communities and disease suppression. We demonstrated that soil microorganisms likely play an important role in disease suppression in soils. In the case of arable soils, it is possible to steer the soil microbial community by applying various organic amendments that can boost specific biological and chemical soil properties that increase plant and soil resilience and/or disease suppression.

## MATERIALS AND METHODS

### Soil and organic amendment characteristics.

Ten different organic amendments were selected as treatments. They were plant- and animal-based and presented a broad range of C/N ratios (from 3.2 to 25.0) and respiration rates (from 3.7 to 38.7 mmol O_2_/kg organic matter [OM]/h) (Table 1). Physicochemical characteristics were measured by Eurofins Agro (Wageningen, The Netherlands) and can be found in supporting information at https://doi.org/10.4121/12971528.

To test the effect of these organic amendments on disease suppression, as well as on several (biological) soil properties, these products were added to two sandy soils. Large batches of bulk soil were sampled from the upper 20 cm of the field, transported to the greenhouse location, stored at room temperature until use. An alluvial sandy soil with a low organic matter content (1.5%) and neutral pH (7.2) was obtained from the experimental fields at Lisse (coordinates: N 52.2552, E 4.5477). The other sandy soil, obtained from the experimental fields at Vredepeel (coordinates: N 51.5417, E 5.8730), had a higher organic matter content (4.0 to 4.3%) and a slightly acidic pH (5.5). More soil characteristics are included in the supporting information at https://doi.org/10.4121/12971528.

The organic amendments were mixed with the soils at 0.2 g N/kg soil to eliminate a possible cofounding effect of nitrogen since N is the main limiting nutrient for (short-term) plant growth. Consequently, the types of organic matter added to the soils differed in their input volumes (Table 1). Granulated products (e.g., manure and seeds) were pulverized, and product with fibers (e.g., plant-3) were chopped into 4-cm fractions prior to application to create a better distribution in the soil. To control for the effect of additional nitrogen on soil disease suppression, two control treatments were included: one with the same amount of nitrogen as the organic amendments was added as calcium nitrate [Ca(NO_3_)_2_] fertilizer (control+N) and another without any additional nitrogen (control). All treatments were prepared in four replicates, and the entire experiment was performed twice (in 2016 and 2017). Soils with the amendments were mixed, put into open plastic bags, and incubated at room temperature (∼20°C) for 3 weeks. This mixed soil was then used for plant disease bioassays and soil biological and physicochemical parameter measurements. A subsample of each amended soil was stored at −80°C for DNA analysis, and the remaining soil was stored in 10% KOH dissolved in methanol at −20°C for ergosterol quantification.

### Plant disease bioassays.

Disease suppressiveness of the amended soils was tested in a bioassay in which the pathogen was artificially inoculated into the amended soils. The fungal pathogen Rhizoctonia solani (AG 2-2IIIB isolate 12-194a; IRS, Bergen op Zoom, The Netherlands) was inoculated into the treated soils, and after disease development was evaluated in susceptible sugar beet seedlings under growth chamber conditions, as described by Postma and Schilder (21). Tanks with an internal size of 4 by 25 by 30 cm were used with prerinsed florist’s foam blocks (water holding capacity ≈ 55%; Van Dillewijn Verpakkingen BV, Aalsmeer, The Netherlands) of 4 by 25 by 17 cm placed at the bottom of each tank. In each tank, 12 cm of treated soil was packed on top of the water-saturated foam blocks. Sugar beet seeds (F763156 with standard seed treatment hymexazol, thiram, and the insecticide Gaucho1; SESVanderHave, Rilland, The Netherlands) were planted in two rows 2 cm deep with a 2-cm distance between seeds at a density of 22 seeds per tank. The soil surface in each tank was watered and covered with plastic foil for 1 week. Then the soil in each tank was inoculated with five oat kernels colonized with R. solani. The inoculation point was set in one of the sides of the tank, 2 cm in front of the first seedling, just below the soil surface. The disease spread, defined as centimeters from the inoculation point to the last seedling in the row with symptoms, was measured twice a week by counting the centimeter to the last seedling displaying damping off or brown-gray lesions on the stem at the soil level. Disease spread data from 21 days after inoculation with the pathogen were used for our study, since a maximum disease spread of 22 cm was reached in the susceptible treatments at this time point. The experiments were carried out with four replicates in a randomized block design, with each replicate soil amendment treatment present in each block. The bioassay was performed in a growth chamber under the following conditions: 23/18°C (day/night), 60% humidity, and a day/night regimen of 8 h of dark and 16 h of light (230 μmol m^−2^ s^−1^ photoactive light; TL280HF) and the soil water potential was automatically regulated at −5 × 10^3^ Pa (soil-water metric potential [pF], 1.7).

Bioassay results from the Vredepeel 2016 soil were excluded in the follow-up data analysis because reduced sugar beet growth was observed in all treatments—possibly due to the application of the herbicide MCPA (2-methyl-4-chlorophenoxyacetic acid) in the field 1 month before the start of the bioassays.

### Biological and physicochemical soil characterization.

Bacterial biomass was determined by confocal laser scanning microscopy (56) and fungal biomass by microscopic counting after staining with fluorescent dyes (57). The concentration of ergosterol, an important sterol in the membrane of most fungi, was measured in the soil samples as an indicator of fungal biomass (58). Hot water extractable carbon (HWC) was measured as the organic carbon present in solution after a 16-h extraction of 4 g of soil in 30 ml water at 80°C. HWC is a labile fraction of organic matter, which originates mainly from microbial exudates and has been proposed as an easily applicable indicator of soil quality (56). Potentially mineralizable nitrogen (PMN)—as an indicator of labile nitrogen that is potentially available for plants—was measured as the increase in ammonium (NH_4_^+^) during 1 week of anaerobic (waterlogged) incubation of 16 g of soil in 40 ml water at 40°C (59, 60). Other physicochemical properties of the amended soil samples were analyzed by the commercial laboratory of Eurofins Agro (Wageningen, The Netherlands). More than 20 nutrient levels in the soils were determined: 13 nutrients (P, K, Mg, Na, B, Cu, Mn, Co, Zn, Se, Si, Mo, and Fe) were measured via the CaCl_2_ extraction method. Total N, S, C, and P were extracted by ammonium lactate, and K, Ca, Mg and Na were extracted via the Cohex method (61).

### DNA extraction, sequencing, and data preprocessing.

DNA was extracted using the MoBio PowerMag soil DNA isolation KF kit (MoBio Laboratories, Inc., Carlsbad, CA, USA). The manufacturer’s protocol was adapted to fit a 4-fold input weight of 1 g soil for each sample. All steps up to the processing of the samples with the King Fisher platform were performed in 5-ml tubes, and volumes of all buffers were proportionally adapted. Lysis was performed in 5-ml MoBio PowerWater DNA bead tubes supplemented with 1 g of 0.1-mm glass beads (MoBio Laboratories, Inc., Carlsbad, CA, USA). In the setup for the King Fisher DNA processing, two technical replicates per sample were used in a 96-well format, and a double binding step was included in the protocol to accommodate all available lysate per sample. The resulting DNA eluates were pooled per sample and stored at −20°C. The DNA concentration was measured with a Pico Green assay (Quant-IT Pico Green dsDNA assay kit; Invitrogen) on a Tecan Infinite M200Pro (Tecan Group, Ltd.). Based on the concentrations, DNA was diluted to 4 ng μl^−1^ with the elution buffer of the DNA extraction kit used as the template for the PCRs. Universal primers E341F (5′-CCTACGGGNGGCWGCAG-3′) and 805R (5′-GACTACHVGGGTATCTAATCC-3′) were used to target the bacterial V3-V4 16S rRNA gene region (62, 63), and primers ITS86F (5′-GTGAATCATCGAATCTTTGAA-3′) and ITS4 (5′-TCCTCCGCTTATTGATATGC-3′) were used for the fungal ITS2 region (64–66). All primers were synthesized (Integrated DNA Technologies, BVBA, Leuven, Belgium) with the universal Illumina MiSeq adapters. Each PCR mixture contained 1× Q5 reaction buffer, 200 μM deoxynucleoside triphosphates (dNTPs), 0.5 μM each primer, 20 ng template DNA, 1 U Q5 Hot Start High-Fidelity DNA polymerase (New England Biolabs, MA, USA), and nuclease-free water to a final volume of 50 μl. The PCR conditions for the target regions 16S V3-V4 and ITS2 were 98°C for 30 s, followed by 15 or 25 cycles of 98°C for 10 s, 55 or 51°C for 30 s and 72°C for 30 s, and a final elongation at 72°C for 2 min in a Veriti Thermo Cycler (Thermo Fisher Scientific, USA). Four replicate PCRs were performed in separate runs for each sample for both primer sets, and replicates were pooled and stored at −20°C. Amplicons were purified and libraries were prepared according to Illumina guidelines (Illumina, San Diego, CA, USA) and paired-end sequenced with 2 × 250 cycles for 16S V3-V4 libraries and 2 × 300 cycles for ITS2 libraries on an Illumina MiSeq platform, and all reads were demultiplexed (Next Generation Sequencing Facilities, Wageningen University & Research, Wageningen, The Netherlands).

Preprocessing of the demultiplexed reads was performed with QIIME2 (67) v.2018.4. For the ITS2 reads, *cutadapt* (68) was used as a plugin in QIIME2, to remove primers and possible read-through in the reads. All raw sequence data were denoised with DADA2 q2 plugin (69). Run-specific quality control and filtering, primer removal for 16S data, merging of paired-end reads, and chimera filtering, resulting in tables with the abundances of exact amplicon sequence variants (ASVs) (70), were performed on each sample and set of unique representative sequences. The resulting DADA2 output was merged for further analysis into a single abundance table. Replicates of sample reads were summed. The ASVs with low abundance (frequency of <0.0005%) were removed. One sample from the 16S data set and two from the ITS data set were removed due to their extremely low library size (see supporting information at https://doi.org/10.4121/12971528). Taxonomy was assigned to the ASVs with the Naive Bayes classifiers plugin in QIIME2 (71, 72). The 16S classifier was pretrained on the extracted 16S V3-V4 region of the Silva 16S/18S database release 132 (73), and the ITS2 classifier was pretrained on the extracted ITS2 region of the Unite database (QIIME release for all eukaryotes v.8.0) with dynamic use of clustering thresholds (74). For the fungal ITS2 database, only ASVs with assignments to the kingdom Fungi were kept. For the bacterial 16S database, ASVs that remained unassigned at the kingdom level or were assigned as mitochondria or chloroplasts were removed.

### Statistical analyses.

In order to test the differences in R. solani disease scores (rs.dis) between the different treatments, a two-way analysis of variance (ANOVA) was performed with the *Anova* function from the CAR R package (75). To indicate the significance between treatments and the reference treatment, a *t* test comparison with Holm correction was applied. The treatment control+N was used as reference in all the comparisons tested. Furthermore, a Spearman correlation analysis was carried out with the biological parameters (PMN, HWC, fungal and bacterial biomass, and ergosterol) and other soil physicochemical data, together with the disease scores, using the *ggscatter* function and Pearson correlation method from the GGPUBR R package (76). Permutation-based *P* values were calculated using the *permcor* function from the RFAST package (77). For the C/N ratio and respiration rate of the products, the average values per treatment were used, as the C/N ratio and respiration rate were only measured once per treatment.

For α diversity, the rarefied abundance data were used. Rarefaction was set at 8,117 sequences for fungi and 6,236 sequences for bacteria. Then richness (observed number of species), the Shannon index, and the inverse of the Simpson index (which takes into account the number of species and how evenly distributed they are) were calculated with the *diversity* function from the VEGAN package (78) and represented with their means in box plots. All data are included, except for Vredepeel 2016, because these data were influenced by herbicide artifact effects (described above).

Normalized data with the *decostand* function (Hellinger normalization) were used for the follow-up analysis, including all data sets, except Vredepeel 2016. In order to see how microbial communities differed between soil type and years, a nonmetric multidimensional scaling (NMDS) analysis with the *metaMDS* function, and a permutational multivariate ANOVA (PERMANOVA) analysis with the *adonis* function, both from the VEGAN package, were performed with a Bray-Curtis dissimilarity index.

To test how microbial communities in the treated soils were related to disease, a canonical correspondence analysis (CCA) using the *cca* function was performed using square root normalized data. The disease spread was used as a constrained variable, and a condition to correct by the factors “year” and “soil” was applied. The top 100 ASVs less associated with disease and more represented in keratin- and chitin-rich treatments were selected from the *cca* object with the function *scores* from the VEGAN package and included in the phylogenetic tree. Loading the abundance data together with sample taxonomy information and metadata, a complex data set object was created with the function *phyloseq* from the PHYLOSEQ R package (79). Subsequently, a phylogenetic tree was created with the function *ggtree* from the GGTREE R package. To study how soil microbial communities are correlated with other soil factors, another CCA was performed. Soil parameters were defined as constrained variables, and they were selected based on their correlation with disease from both the linear model (*lm*) and correlation (*corr*) analyses. The factors “soil” and “year” were included in the model as conditions. An ANOVA of the CCA object per treatment type (keratin and chitin rich) was performed to test for significant relationships with the different soil parameters. From the CCA object, the top 260 ASVs were selected from the *scores* function output to estimate the number of ASVs in each family category for the suppressive treatments and presented in a bar plot.

To study how the fungal and bacterial abundances related to each other in the disease-suppressive amended soils, a Spearman correlation analysis between bacteria and fungi for proportional abundance was performed. Only the keratin- and chitin-rich amendments were included in the analysis. Both bacteria and fungi were filtered, and taxa without at least 50 occurrences were removed. A Spearman rank correlation with an associated *P* value was calculated between each combination of bacteria and fungi. The *P* values were adjusted for multiple comparison with a false-discovery rate (FDR), which was calculated with the Benjamini-Hochberg adjustment. Edges with an FDR of <0.05 and their associated nodes were included in the network diagram.

### Data availability.

All sequences have been submitted to the NCBI database under BioProject no. PRJNA657959.

## Supplementary Material

Download

## References

[1] Oerke E-C. 2006. Crop losses to pests. J Agric Sci 144:31–43. 10.1017/S0021859605005708.

[2] Savary S, Willocquet L, Pethybridge SJ, Esker P, McRoberts N, Nelson A. 2019. The global burden of pathogens and pests on major food crops. Nat Ecol Evol 3:430–439. 10.1038/s41559-018-0793-y. 30718852

[3] Gómez Expósito R, de Bruijn I, Postma J, Raaijmakers JM. 2017. Current insights into the role of rhizosphere bacteria in disease suppressive soils. Front Microbiol 8:2529. 10.3389/fmicb.2017.02529. 29326674 PMC5741648

[4] Schlatter D, Kinkel L, Thomashow L, Weller D, Paulitz T. 2017. Disease suppressive soils: new insights from the soil microbiome. Phytopathology 107:1284–1297. 10.1094/PHYTO-03-17-0111-RVW. 28650266

[5] Larkin RP. 2015. Soil health paradigms and implications for disease management. Annu Rev Phytopathol 53:199–221. 10.1146/annurev-phyto-080614-120357. 26002292

[6] Bonanomi G, Lorito M, Vinale F, Woo SL. 2018. Organic amendments, beneficial microbes, and soil microbiota: toward a unified framework for disease suppression. Annu Rev Phytopathol 56:1–20. 10.1146/annurev-phyto-080615-100046. 29768137

[7] De LI, Palacios JG. 2013. European Union (EU) end of waste regulation: requirements for input materials and compost quality for sludge and other biodegradable wastes. J Residuals Sci Technol 10:139–146.

[8] Alvarenga P, Palma P, Mourinha C, Farto M, Dôres J, Patanita M, Cunha-Queda C, Natal-da-Luz T, Renaud M, Sousa JP. 2017. Recycling organic wastes to agricultural land as a way to improve its quality: a field study to evaluate benefits and risks. Waste Management 61:582–592. 10.1016/j.wasman.2017.01.004. 28089401

[9] Abbott LK, Macdonald LM, Wong MTF, Webb MJ, Jenkins SN, Farrell M. 2018. Potential roles of biological amendments for profitable grain production—a review. Agric Ecosyst Environ 256:34–50. 10.1016/j.agee.2017.12.021.

[10] Noble R, Coventry E. 2005. Suppression of soil-borne plant diseases with composts: a review. Biocontrol Sci Technol 15:3–20. 10.1080/09583150400015904.

[11] Termorshuizen AJ, van Rijn E, van der Gaag DJ, Alabouvette C, Chen Y, Lagerlöf J, Malandrakis AA, Paplomatas EJ, Rämert B, Ryckeboer J, Steinberg C, Zmora-Nahum S. 2006. Suppressiveness of 18 composts against 7 pathosystems: variability in pathogen response. Soil Biol Biochem 38:2461–2477. 10.1016/j.soilbio.2006.03.002.

[12] Bonanomi G, Antignani V, Capodilupo M, Scala F. 2010. Identifying the characteristics of organic soil amendments that suppress soilborne plant diseases. Soil Biol Biochem 42:136–144. 10.1016/j.soilbio.2009.10.012.

[13] Akhtar M. 1993. Utilisation of plant-origin waste materials for the control of plant-parasitic nematodes. Bioresour Technol 46:255–257. 10.1016/0960-8524(93)90129-Y.

[14] Hallmann J, Rodríguez-Kábana R, Kloepper JW. 1999. Chitin-mediated changes in bacterial communities of the soil, rhizosphere and within roots of cotton in relation to nematode control. Soil Biol Biochem 31:551–560. 10.1016/S0038-0717(98)00146-1.

[15] Korthals GW, Thoden TC, van den Berg W, Visser JHM. 2014. Long-term effects of eight soil health treatments to control plant-parasitic nematodes and *Verticillium dahliae* in agro-ecosystems. Appl Soil Ecol 76:112–123. 10.1016/j.apsoil.2013.12.016.

[16] Henis Y, Sneh B, Katan J. 1967. Effect of organic amendments on *Rhizoctonia* and accompanying microflora in soil. Can J Microbiol 13:643–650. 10.1139/m67-085. 6035142

[17] Postma J, Scheper RWA, Schilder MT. 2010. Effect of successive cauliflower plantings and *Rhizoctonia solani* AG 2-1 inoculations on disease suppressiveness of a suppressive and a conducive soil. Soil Biol Biochem 42:804–812. 10.1016/j.soilbio.2010.01.017.

[18] Inderbitzin P, Ward J, Barbella A, Solares N, Izyumin D, Burman P, Chellemi DO, Subbarao KV. 2018. Soil microbiomes associated with *Verticillium* wilt-suppressive broccoli and chitin amendments are enriched with potential biocontrol agents. Phytopathology 108:31–43. 10.1094/PHYTO-07-17-0242-R. 28876209

[19] Akhtar M, Alam MM. 1993. Utilization of waste materials in nematode control: a review. Bioresour Technol 45:1–7. 10.1016/0960-8524(93)90134-W.

[20] Lazarovits G. 2010. Managing soilborne disease of potatoes using ecologically based approaches. Am J Pot Res 87:401–411. 10.1007/s12230-010-9157-0.

[21] Postma J, Schilder MT. 2015. Enhancement of soil suppressiveness against *Rhizoctonia solani* in sugar beet by organic amendments. Appl Soil Ecol 94:72–79. 10.1016/j.apsoil.2015.05.002.

[22] Xiong W, Guo S, Jousset A, Zhao Q, Wu H, Li R, Kowalchuk GA, Shen Q. 2017. Bio-fertilizer application induces soil suppressiveness against Fusarium wilt disease by reshaping the soil microbiome. Soil Biol Biochem 114:238–247. 10.1016/j.soilbio.2017.07.016.

[23] Oka Y. 2010. Mechanisms of nematode suppression by organic soil amendments—a review. Appl Soil Ecol 44:101–115. 10.1016/j.apsoil.2009.11.003.

[24] Scotti R, Bonanomi G, Scelza R, Zoina A, Rao MA. 2015. Organic amendments as sustainable tool to recovery fertility in intensive agricultural systems. J Soil Sci Plant Nutr 15:333–352. 10.4067/S0718-95162015005000031.

[25] Liu Z, Rong Q, Zhou W, Liang G. 2017. Effects of inorganic and organic amendment on soil chemical properties, enzyme activities, microbial community and soil quality in yellow clayey soil. PLoS One 12:e0172767. 10.1371/journal.pone.0172767. 28263999 PMC5338777

[26] Cretoiu MS, Korthals GW, Visser JHM, van Elsas JD. 2013. Chitin amendment increases soil suppressiveness toward plant pathogens and modulates the actinobacterial and oxalobacteraceal communities in an experimental agricultural field. Appl Environ Microbiol 79:5291–5301. 10.1128/AEM.01361-13. 23811512 PMC3753968

[27] Cesarano G, De Filippis F, La Storia A, Scala F, Bonanomi G. 2017. Organic amendment type and application frequency affect crop yields, soil fertility and microbiome composition. Appl Soil Ecol 120:254–264. 10.1016/j.apsoil.2017.08.017.

[28] Clocchiatti A, Hannula SE, van den Berg M, Korthals G, de Boer W. 2020. The hidden potential of saprotrophic fungi in arable soil: patterns of short-term stimulation by organic amendments. Appl Soil Ecol 147:103434. 10.1016/j.apsoil.2019.103434.

[29] Gomez-Exposito R. 2017. Microbiome dynamics of disease suppressive soils. Wageningen University, Wageningen, The Netherlands.

[30] Schippers B, Palm LC. 1973. Ammonia, a fungistatic volatile in chitin-amended soil. Netherlands J Plant Pathol 79:279–281. 10.1007/BF01976677.

[31] Coelho L, Reis M, Guerrero C, Dionísio L. 2020. Use of organic composts to suppress bentgrass diseases in Agrostis stolonifera. Biological Control 141:104154. 10.1016/j.biocontrol.2019.104154.

[32] Bhange K, Chaturvedi V, Bhatt R. 2016. Ameliorating effects of chicken feathers in plant growth promotion activity by a keratinolytic strain of Bacillus subtilis PF1. Bioresour Bioprocess 3:13. 10.1186/s40643-016-0091-y.

[33] Tamreihao K, Mukherjee S, Khunjamayum R, Devi LJ, Asem RS, Ningthoujam DS. 2019. Feather degradation by keratinolytic bacteria and biofertilizing potential for sustainable agricultural production. J Basic Microbiol 59:4–13. 10.1002/jobm.201800434. 30353928

[34] Calin M, Raut I, Arsene ML, Capra L, Gurban AM, Doni M, Jecu L. 2019. Applications of fungal strains with keratin-degrading and plant growth promoting characteristics. Agronomy 9:543. 10.3390/agronomy9090543.

[35] Swiontek Brzezinska M, Jankiewicz U, Burkowska A, Walczak M. 2014. Chitinolytic microorganisms and their possible application in environmental protection. Curr Microbiol 68:71–81. 10.1007/s00284-013-0440-4. 23989799 PMC3889922

[36] Kacprzak M, Malina G. 2005. The tolerance and Zn^2+^, Ba^2+^ and Fe^3+^ accumulation by *Trichoderma atroviride* and *Mortierella exigua* isolated from contaminated soil. Can J Soil Sci 85:283–290. 10.4141/S04-018.

[37] Li F, Chen L, Redmile-Gordon M, Zhang J, Zhang C, Ning Q, Li W. 2018. Mortierella elongata’s roles in organic agriculture and crop growth promotion in a mineral soil. Land Degrad Dev 29:1642–1651. 10.1002/ldr.2965.

[38] Tamayo-Vélez Á, Osorio NW. 2018. Soil fertility improvement by litter decomposition and inoculation with the fungus *Mortierella* sp. in avocado plantations of Colombia. Commun Soil Sci Plant Anal 49:139–147. 10.1080/00103624.2017.1417420.

[39] Lange L, Huang Y, Busk PK. 2016. Microbial decomposition of keratin in nature—a new hypothesis of industrial relevance. Appl Microbiol Biotechnol 100:2083–2096. 10.1007/s00253-015-7262-1. 26754820 PMC4756042

[40] Hemissi I, Mabrouk Y, Mejri S, Saidi M, Sifi B. 2013. Enhanced defence responses of chickpea plants against *Rhizoctonia solani* by pre-inoculation with rhizobia. J Phytopathol 161:412–418. 10.1111/jph.12071.

[41] He W-J, Shi M-M, Yang P, Huang T, Zhao Y, Wu A-B, Dong W-B, Li H-P, Zhang J-B, Liao Y-C. 2020. A quinone-dependent dehydrogenase and two NADPH-dependent aldo/keto reductases detoxify deoxynivalenol in wheat via epimerization in a *Devosia* strain. Food Chem 321:126703. 10.1016/j.foodchem.2020.126703. 32247890

[42] Ofek M, Hadar Y, Minz D. 2012. Ecology of root colonizing *Massilia* (Oxalobacteraceae). PLoS One 7:e40117. 10.1371/journal.pone.0040117. 22808103 PMC3394795

[43] Bach E, Cannavan FS, Duarte FRS, Taffarel JAS, Tsai SM, Brandelli A. 2011. Characterization of feather-degrading bacteria from Brazilian soils. Int Biodeterioration Biodegradation 65:102–107. 10.1016/j.ibiod.2010.07.005.

[44] Kraková L, Šoltys K, Puškárová A, Bučková M, Jeszeová L, Kucharík M, Budiš J, Orovčík L, Szemes T, Pangallo D. 2018. The microbiomes of a XVIII century mummy from the castle of Krásna Hôrka (Slovakia) and its surrounding environment. Environ Microbiol 20:3294–3308. 10.1111/1462-2920.14312. 30051567

[45] Herzog B, Overy DP, Haltli B, Kerr RG. 2016. Discovery of keratinases using bacteria isolated from marine environments. Syst Appl Microbiol 39:49–57. 10.1016/j.syapm.2015.10.004. 26607323

[46] van der Heijden MGA, Wagg C. 2013. Soil microbial diversity and agro-ecosystem functioning. Plant Soil 363:1–5. 10.1007/s11104-012-1545-4.

[47] Mendes LW, Tsai SM, Navarrete AA, de Hollander M, van Veen JA, Kuramae EE. 2015. Soil-borne microbiome: linking diversity to function. Microb Ecol 70:255–265. 10.1007/s00248-014-0559-2. 25586384

[48] Schipper LA, Sparling GP. 2000. Performance of soil condition indicators across taxonomic groups and land uses. Soil Sci Soc Am J 64:300–311. 10.2136/sssaj2000.641300x.

[49] van Elteren JT, Woroniecka UD, Kroon KJ. 1998. Accumulation and distribution of selenium and cesium in the cultivated mushroom *Agaricus bisporus*—a radiotracer-aided study. Chemosphere 36:1787–1798. 10.1016/S0045-6535(97)10064-9.

[50] Vetter J, Lelley J. 2004. Selenium level of the cultivated mushroom *Agaricus bisporus*. Acta Alimentaria 33:297–301. 10.1556/AAlim.33.2004.3.10.

[51] Saikia R, Varghese S, Singh BP, Arora DK. 2009. Influence of mineral amendment on disease suppressive activity of *Pseudomonas fluorescens* to *Fusarium* wilt of chickpea. Microbiol Res 164:365–373. 10.1016/j.micres.2007.05.001. 17604612

[52] Malandrakis AA, Kavroulakis N, Chrysikopoulos CV. 2019. Use of copper, silver and zinc nanoparticles against foliar and soil-borne plant pathogens. Sci Total Environ 670:292–299. 10.1016/j.scitotenv.2019.03.210. 30903901

[53] Busk PK, Lange L. 2015. Classification of fungal and bacterial lytic polysaccharide monooxygenases. BMC Genomics 16:368. 10.1186/s12864-015-1601-6. 25956378 PMC4424831

[54] Aachmann FL, Sørlie M, Skjåk-Bræk G, Eijsink VGH, Vaaje-Kolstad G. 2012. NMR structure of a lytic polysaccharide monooxygenase provides insight into copper binding, protein dynamics, and substrate interactions. Proc Natl Acad Sci U S A 109:18779–18784. 10.1073/pnas.1208822109. 23112164 PMC3503203

[55] Shamshina JL, Kelly A, Oldham T, Rogers RD. 2020. Agricultural uses of chitin polymers. Environ Chem Lett 18:53–60. 10.1007/s10311-019-00934-5.

[56] Bloem J, Veninga M, Shepherd J. 1995. Fully automatic determination of soil bacterium numbers, cell volumes, and frequencies of dividing cells by confocal laser scanning microscopy and image analysis. Appl Environ Microbiol 61:926–936. 10.1128/AEM.61.3.926-936.1995. 16534976 PMC1388375

[57] Bloem J, Vos A. 2004. Fluorescent staining of microbes for total direct counts, p 861–874. *In* Kokalchuk GA, de Bruijn FJ, Head IM, Akkermans AD, van Elsas JD (ed), Molecular microbial ecology manual, 2nd ed. Springer, New York, NY.

[58] Ridder-Duine ASD, Smant W, der Wal AV, Veen JAV, Boer WD. 2006. Evaluation of a simple, non-alkaline extraction protocol to quantify soil ergosterol. Pedobiologia 50:293–300. 10.1016/j.pedobi.2006.03.004.

[59] Canali S, Benedetti A. 2006. Soil nitrogen mineralization, p 127–135. *In* Bloem J, Hopkins DW, Benedetti A (ed), Microbiological methods for assessing soil quality. CABI, Wallingford, United Kingdom.

[60] Keeney DR, Nelson DW. 2015. Nitrogen—inorganic forms, p 643–698. *In* Page AL (ed), Methods of soil analysis. John Wiley & Sons, Ltd, Hoboken, NJ.

[61] Houba VJG, Novozamsky I, Lexmond TM, van der Lee JJ. 1990. Applicability of 0.01 M CaCl_2_ as a single extraction solution for the assessment of the nutrient status of soils and other diagnostic purposes. Commun Soil Sci Plant Anal 21:2281–2290. 10.1080/00103629009368380.

[62] Herlemann DP, Labrenz M, Jürgens K, Bertilsson S, Waniek JJ, Andersson AF. 2011. Transitions in bacterial communities along the 2000 km salinity gradient of the Baltic Sea. ISME J 5:1571–1579. 10.1038/ismej.2011.41. 21472016 PMC3176514

[63] Klindworth A, Pruesse E, Schweer T, Peplies J, Quast C, Horn M, Glöckner FO. 2013. Evaluation of general 16S ribosomal RNA gene PCR primers for classical and next-generation sequencing-based diversity studies. Nucleic Acids Res 41:e1. 10.1093/nar/gks808. 22933715 PMC3592464

[64] White TJ, Bruns T, Lee S, Taylor J, Innis MA, Gelfand DH, Sninsky J. 1990. Amplification and direct sequencing of fungal ribosomal RNA genes for phylogenetics, p 315–322. *In* White TJ (ed), PCR protocols. A guide to methods and applications. Academic Press, Inc, Waltham, MA.

[65] Turenne CY, Sanche SE, Hoban DJ, Karlowsky JA, Kabani AM. 1999. Rapid identification of fungi by using the ITS2 genetic region and an automated fluorescent capillary electrophoresis system. J Clin Microbiol 37:1846–1851. 10.1128/JCM.37.6.1846-1851.1999. 10325335 PMC84966

[66] Beeck MOD, Lievens B, Busschaert P, Declerck S, Vangronsveld J, Colpaert JV. 2014. Comparison and validation of some ITS primer pairs useful for fungal metabarcoding studies. PLoS One 9:e97629. 10.1371/journal.pone.0097629. 24933453 PMC4059633

[67] Bolyen E, Rideout JR, Dillon MR, Bokulich NA, Abnet CC, Al-Ghalith GA, Alexander H, Alm EJ, Arumugam M, Asnicar F, Bai Y, Bisanz JE, Bittinger K, Brejnrod A, Brislawn CJ, Brown CT, Callahan BJ, Caraballo-Rodríguez AM, Chase J, Cope EK, Da Silva R, Diener C, Dorrestein PC, Douglas GM, Durall DM, Duvallet C, Edwardson CF, Ernst M, Estaki M, Fouquier J, Gauglitz JM, Gibbons SM, Gibson DL, Gonzalez A, Gorlick K, Guo J, Hillmann B, Holmes S, Holste H, Huttenhower C, Huttley GA, Janssen S, Jarmusch AK, Jiang L, Kaehler BD, Kang KB, Keefe CR, Keim P, Kelley ST, Knights D, . 2019. Reproducible, interactive, scalable and extensible microbiome data science using QIIME 2. Nat Biotechnol 37:852–857. 10.1038/s41587-019-0209-9. 31341288 PMC7015180

[68] Martin M. 2011. Cutadapt removes adapter sequences from high-throughput sequencing reads. EMBnet J 17:10–12. 10.14806/ej.17.1.200.

[69] Callahan BJ, McMurdie PJ, Rosen MJ, Han AW, Johnson AJA, Holmes SP. 2016. DADA2: high-resolution sample inference from Illumina amplicon data. Nat Methods 13:581–583. 10.1038/nmeth.3869. 27214047 PMC4927377

[70] Callahan BJ, McMurdie PJ, Holmes SP. 2017. Exact sequence variants should replace operational taxonomic units in marker-gene data analysis. ISME J 11:2639–2643. 10.1038/ismej.2017.119. 28731476 PMC5702726

[71] Pedregosa F, Varoquaux G, Gramfort A, Michel V, Thirion B, Grisel O, Blondel M, Prettenhofer P, Weiss R, Dubourg V, Vanderplas J, Passos A, Cournapeau D, Brucher M, Perrot M, Duchesnay É. 2011. Scikit-learn: machine learning in Python. J Machine Learning Res 12:2825–2830.

[72] Bokulich NA, Kaehler BD, Rideout JR, Dillon M, Bolyen E, Knight R, Huttley GA, Gregory Caporaso J. 2018. Optimizing taxonomic classification of marker-gene amplicon sequences with QIIME 2’s q2-feature-classifier plugin. Microbiome 6:90. 10.1186/s40168-018-0470-z. 29773078 PMC5956843

[73] Quast C, Pruesse E, Yilmaz P, Gerken J, Schweer T, Yarza P, Peplies J, Glöckner FO. 2013. The SILVA ribosomal RNA gene database project: improved data processing and web-based tools. Nucleic Acids Res 41:D590–D596. 10.1093/nar/gks1219. 23193283 PMC3531112

[74] Nilsson RH, Larsson K-H, Taylor AFS, Bengtsson-Palme J, Jeppesen TS, Schigel D, Kennedy P, Picard K, Glöckner FO, Tedersoo L, Saar I, Kõljalg U, Abarenkov K. 2019. The UNITE database for molecular identification of fungi: handling dark taxa and parallel taxonomic classifications. Nucleic Acids Res 47:D259–D264. 10.1093/nar/gky1022. 30371820 PMC6324048

[75] Fox J, Weisberg S. 2011. An R companion to applied regression. Sage Publications, Los Angeles, CA.

[76] Kassambara A. 2018. GGPUBR: “ggplot” Based Publication Ready Plots. R Package version 0.1.7. http://www.sthda.com/english/articles/24-ggpubr-publication-ready-plots/.

[77] Papadakis M, Tsagris M, Dimitriadis M, Fafalios S, Tsamardinos I, Fasiolo M, Borboudakis G, Burkardt J, Zou C, Lakiotaki K, Chatzipantsiou C. 2019. Package ‘Rfast’: a collection of efficient and extremely fast R functions. https://cran.r-project.org/web/packages/Rfast/Rfast.pdf.

[78] Oksanen J, Blanchet FG, Kindt R, Legendre P, Minchin PR, O’Hara RB, Simpson GL, Solymos P, Stevens MHL, Wagner H. 2015. Package ‘vegan’: community ecology package. Version 2.3-2. https://cran.r-project.org/web/packages/vegan/index.html.

[79] McMurdie PJ, Holmes S. 2013. phyloseq: an R package for reproducible interactive analysis and graphics of microbiome census data. PLoS One 8:e61217. 10.1371/journal.pone.0061217. 23630581 PMC3632530

