# Supplementary material for: Chitin- and Keratin-Rich Soil Amendments Suppress Rhizoctonia solani Disease via Changes to the Soil Microbial Community
Source: Appl Environ Microbiol. 2021 May 11;87(11):e00318-21. doi: 10.1128/AEM.00318-21 (PMC8208141; doi:10.1128/AEM.00318-21)
Supplement: Download [file AEM.00318-21_aem.00318-21-s0001.pdf]

Figure S1. Biological soil parameters.

(a) Potential mineralizable nitrogen (PMN), Hot water extractable carbon (HWC), ergosterol, fungal biomass and bacterial biomass in the different treatments are shown. Asterisks represent the *P*-adjusted values from pairwise t.test comparisons ('holm' correction) using Control+N as a reference.

(b) Results from ANOVA analysis that tests the effect of the factors year, soil and treatment in the different soil parameters. **P*<0.05; ***P*<0.01; ****P*<0.001; ns: not significant.

(a)

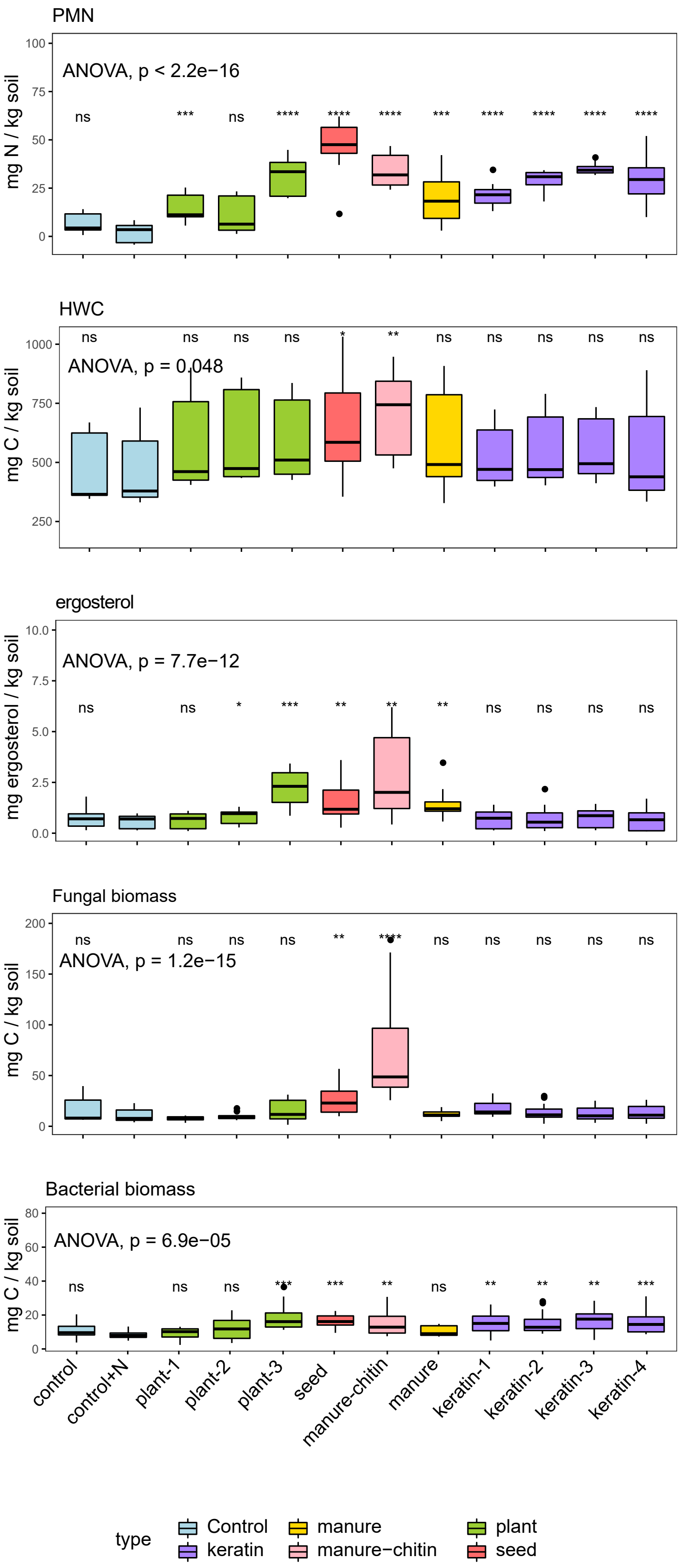

(b)

PMN

Factor	Sum Sq	Df	F	Pr(>F)	
treatment	23131	11	53.0	2.20E-16	***
soil	1071	1	27.0	9.80E-07	***
year	34	1	0.9	0.35789	
treatment:soil	931	11	2.1	0.02368	*
treatment:year	2453	11	5.6	4.50E-07	***
soil:year	0				
treatment:soil:year	0				
Residuals	4287	108			

HWC

Factor	Sum Sq	Df	F	Pr(>F)	
treatment	558792	11	12.5	6.43E-15	***
soil	2127722	1	523.7	2.20E-16	***
year	6970	1	1.7	0.193047	
treatment:soil	119394	11	2.7	0.004527	**
treatment:year	122450	11	2.7	0.003654	**
soil:year	0				
treatment:soil:year	0				
Residuals	438791	108			

ergosterol

Factor	Sum Sq	Df	F	Pr(>F)	
treatment	70	11	28.7	2.20E-16	***
soil	9	1	41.6	3.31E-09	***
year	38	1	172.3	2.20E-16	***
treatment:soil	2	11	0.7	0.7432	
treatment:year	26	11	10.8	4.02E-13	***
soil:year	0				
treatment:soil:year	0				
Residuals	24	107			

bacterial biomass

Factor	Sum Sq	Df	F	Pr(>F)	
treatment	1491	11	5.3	1.23E-06	***
soil	288	1	11.2	0.001109	**
year	413	1	16.1	0.00011	***
treatment:soil	234	11	0.8	0.611829	
treatment:year	186	11	0.7	0.773036	
soil:year	0				
treatment:soil:year	0				
Residuals	2770	108			

fungal biomass

Factor	Sum Sq	Df	F	Pr(>F)	
treatment	42971	11	46.6	2.20E-16	***
soil	274	1	3.3	0.07337	.
year	7544	1	90.0	6.87E-16	***
treatment:soil	501	11	0.5	0.86933	
treatment:year	12900	11	14.0	2.43E-16	***
soil:year	0				
treatment:soil:year	0				
Residuals	9053	108			

Table S1. Biological and physico-chemical parameters measured in the present study.
a: available for plants and microorganisms; t: total.

name	description	units
B.t	boron	mmol+/kg
C:N	carbon/nitrogen ratio	-
Ca.a	calcium available	mmol+/l
Ca.t	calcium total	mmol+/kg
CaCO3	carbonic lime	%
CEC	cation exchange capacity	mmol+/kg
Co.a	cobalt available	µg/kg
Cu.a	copper available	µg/kg
K.a	potasium available	mg/kg
K.t	potasium total	mmol+/kg
Lutum	lutum	%
Mg.t	magnesium	mmol+/kg
Mn.t	manganesum	mmol+/kg
Mo.a	molybdenum available	µg/kg
N.a	free nitrogen supplied by soil	mg/kg
N.t	total nitrogen	mg/kg
Na.t	sodium	mmol+/kg
OM	organic matter	%
P.a	phosphorous available	mg/kg
P.t	Total phosphorous	mg P_2O_5/l
pH	pH	-
S.a	available sulfur	mg/kg
S.t	sulfur total	mg/kg
sand	sand (>50 um)	%
Se.a	selenium available	µg/kg
silica	silicon available	µg/kg
silt	silt (2-5 um)	%
Zn.a	zinc available	µg/kg
fungi	fungal biomass	mg C/kg
bacteria	bacterial biomass	mg C/kg
ergosterol	ergosterol	mg/kg
PMN	Potentially Mineralizable Nitrogen	mg N/kg
HWC	hot water extractable carbon	mg C/kg
Rs.dis	*Rhizoctonia solani* disease spread	cm*

*from inoculation point

Figure S2. Physico-chemical soil parameters in the different treatments.
Asterisks represent the *P*-adjusted values from pairwise t.test comparisons ('holm' correction) using Control+N as reference. **P*<0.05; ***P*<0.01; ****P*<0.001; ns: not significant.
Data from Vredepeel 2016 are removed from the analysis. Only the variables that were significantly different in at least one treatment are shown.

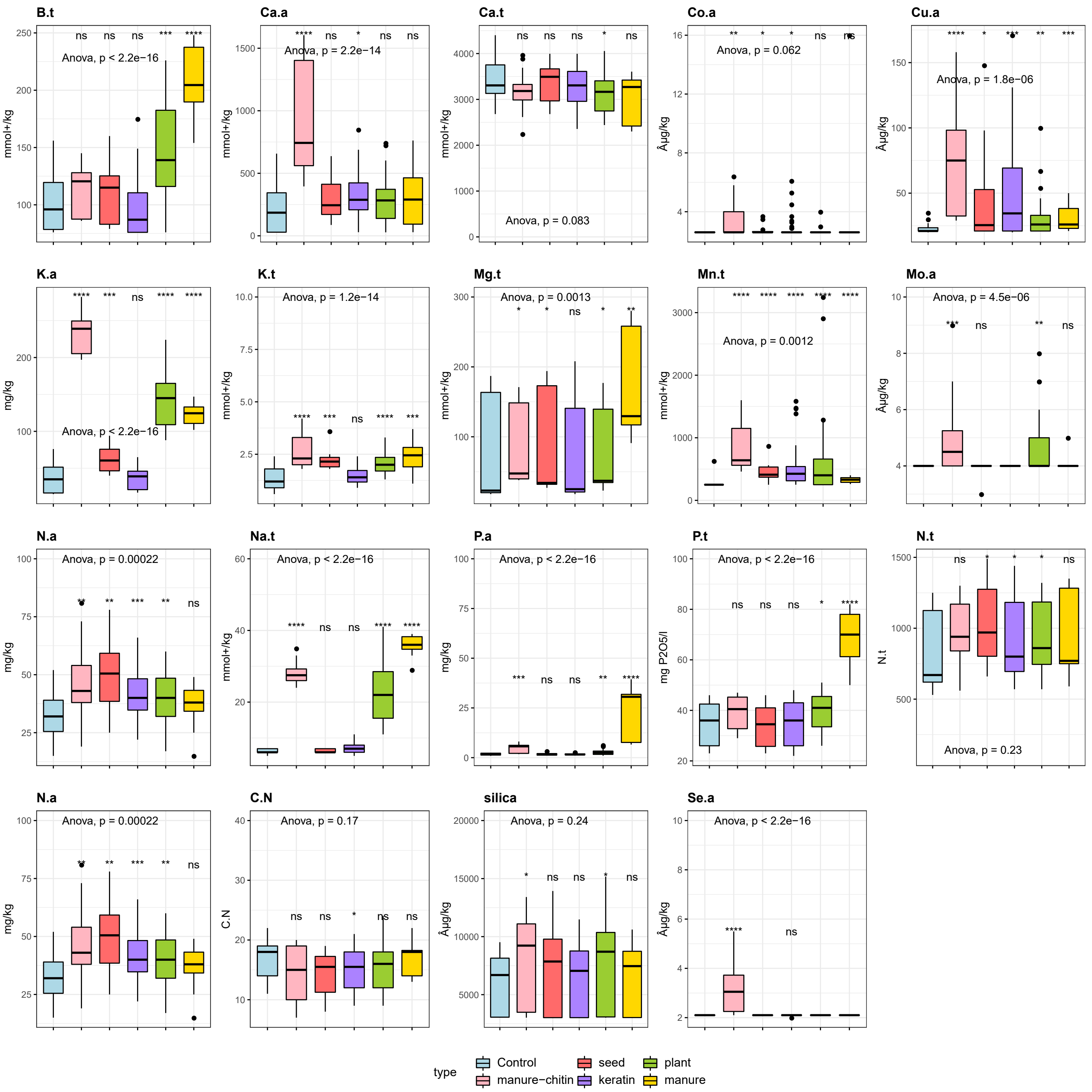

Figure S3. Microbial communities in the different soils.
Multivariate NMDS analysis applying 'Bray-Curtis' distance that shows fungal and bacterial communities are different between soils and also to a lesser extend between years. Table shows adonis analysis for the soil/year differences.

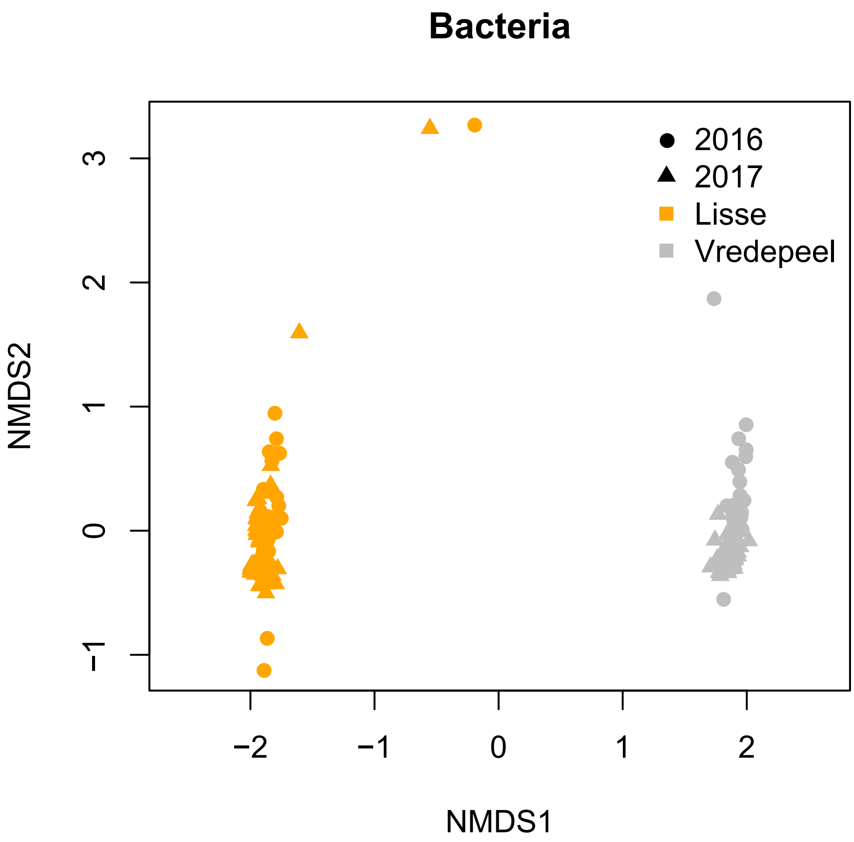

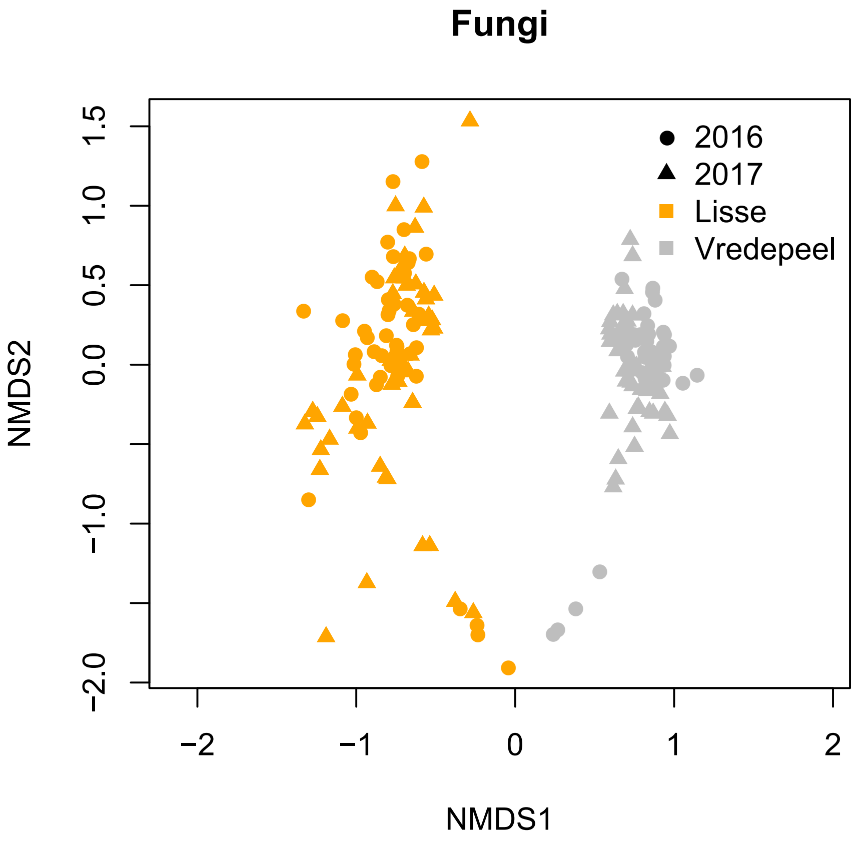

factor	group	Df	Sums Sq	Mean Sq	F	R^2	Pr(<F)
soil	Bacteria	1	23.10	23.10	125.11	0.40	0.001***
soil	Fungi	1	14.75	14.75	58.96	0.24	0.001***
year	Bacteria	1	2.45	2.45	8.35	0.04	0.001***
year	Fungi	1	2.75	2.75	8.78	0.04	0.001***

Figure S4. Number of taxa groups found in the study.
(a) Bacteria (b) Fungi. Top 50 taxa with the highest number of different ASVs are represented.
Data based on total number of taxa in the study. Showing only Families with more than 50 ASVs (n>50).

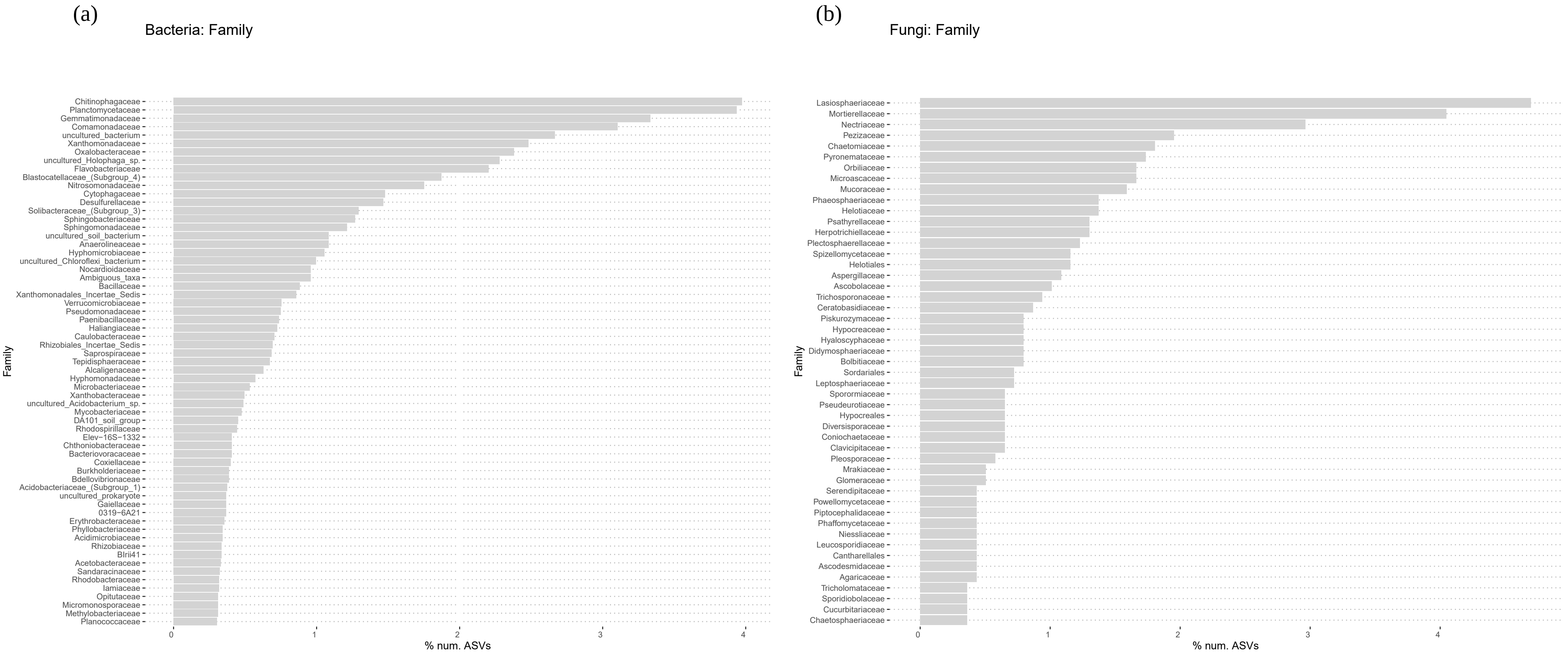

Figure S5. Network plot.
For each edge a Spearman correlation and an associated *P*-value was calculated. Only edges with a FDR below 0.05 are included in the diagram. Red and blue arrows indicate negative and positive Spearman correlations, respectively. Blue and yellow nodes represent bacteria and fungi respectively. All keratin- and chitin-rich amendments samples are included in the analysis.

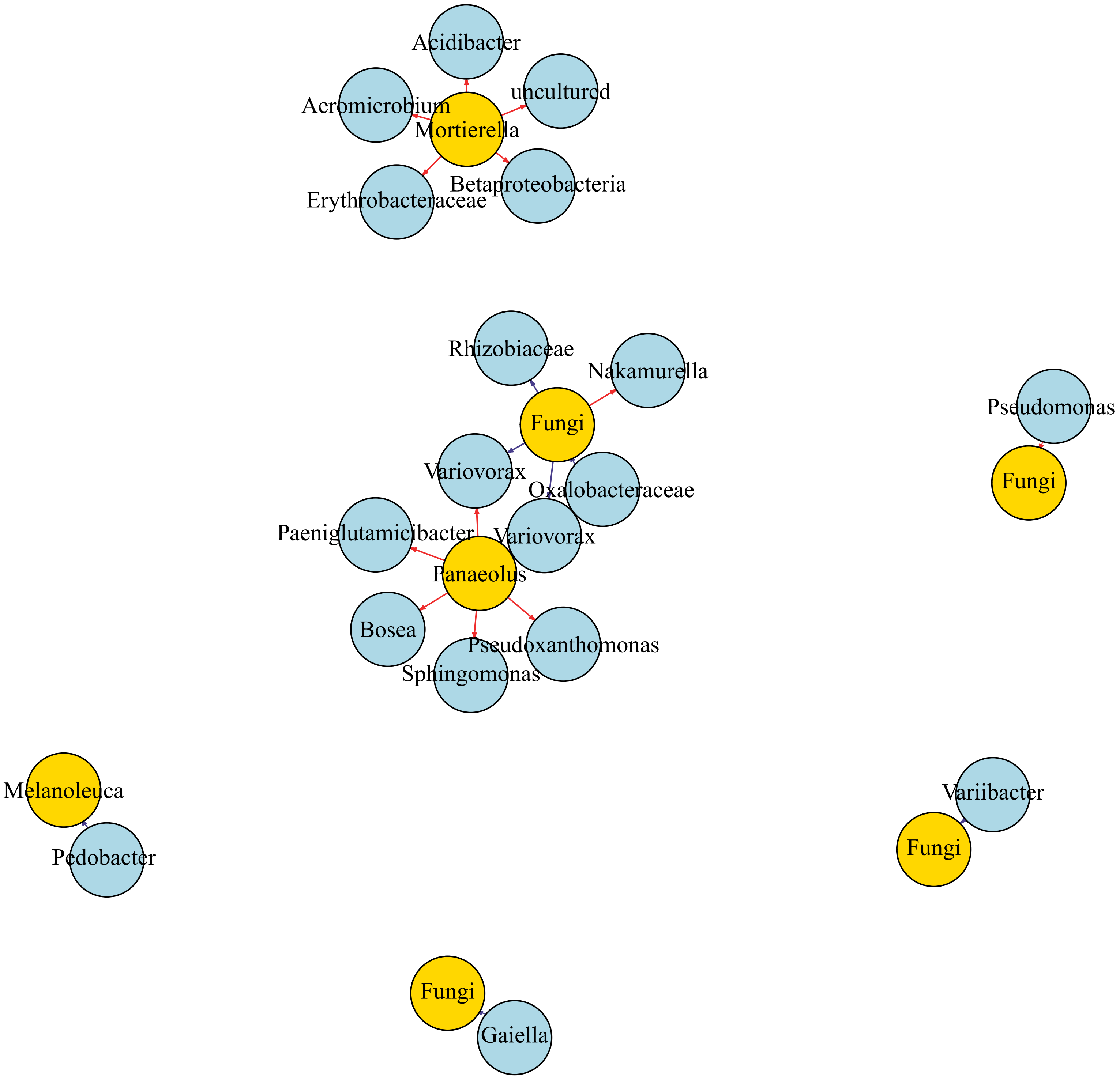

Table S2. Data output from a ANOVA test with the RDA object.

Table shows the significance between the different chemical and physical properties that are related to the bacterial (a) and fungal (b) populations in chitin- and keratin-rich amendments. Differences show if a factor is associated (negatively/positively) with the microbial communities in these treatments. *$P<0.05$; **$P<0.01$; ***$P<0.001$.

BACTERIA						FUNGI					
Keratin-rich						*Keratin-rich*					
variable	Df	Variance	F	Pr(>F)		variable	Df	Variance	F	Pr(>F)	
Zn.a	1	0.01474	3.3438	0.001	***	P.a	1	0.01008	2.6137	0.002	**
P.a	1	0.00877	1.9903	0.001	***	Mn.t	1	0.00813	2.1094	0.01	**
Cu.a	1	0.00778	1.764	0.006	**	Cu.a	1	0.00702	1.8203	0.037	*
Mn.t	1	0.00696	1.5794	0.019	*						
Ca.a	1	0.00621	1.4077	0.05	*						
Chitin-rich						*Chitin-rich*					
variable	Df	Variance	F	Pr(>F)		*No significantly different variables found*					
Cu.a	1	0.06324	3.5155	0.009	**						
Se	1	0.05359	2.7917	0.039	*						
Co.a	1	0.05125	2.63	0.03	*						

Supplementary Information (Data files)
All the following files have been uploaded in 4TU database: doi 10.4121/12971528.

Data1: products_properties.csv

Data2: Bacteria_taxonomy_16S.tsv

Data3: Fungi_taxonomy_unite_ITS2.tsv

Data4: Bacteria_ASV_keratin-chitin_from_CCA.csv

Data5: Fungi_ASV_keratin-chitin_from_CCA.csv

Data6: Bacteria_table_counts_16S.tsv

Data7: Fungi_table_counts_ITS.tsv

Data8: Bacteria_metadata_16S.tsv

Data9: Fungi_metadata_ITS.csv

Data10: Bacteria_soil_parameters.csv

Data11: Fungi_soil_parameters.csv

Data12: Rank_list_species_CCA.csv

statistics_2020.R

README_Andreo-Jimenez_et_al.txt
